# Sirtuin 5 depletion impairs mitochondrial function in human proximal tubular epithelial cells

**DOI:** 10.1038/s41598-021-94185-6

**Published:** 2021-07-30

**Authors:** Timo N. Haschler, Harry Horsley, Monika Balys, Glenn Anderson, Jan-Willem Taanman, Robert J. Unwin, Jill T. Norman

**Affiliations:** 1 Department of Renal Medicine, UCL Medical School, Royal Free Campus, 2nd floor, Rowland Hill Street, London, NW3 2PF UK; 2 Early Clinical Development, Research and Early Development, Cardiovascular, Renal and Metabolism (CVRM), BioPharmaceuticals R&D, AstraZeneca, Cambridge, UK; 3 Department of Clinical and Movement Neurosciences, UCL Queen Square Institute of Neurology, Royal Free Campus, London, UK; 4 Department of Histopathology, Great Ormond Street Hospital, London, UK

**Keywords:** Biochemistry, Cell biology, Physiology, Diseases, Molecular medicine, Nephrology

## Abstract

Ischemia is a major cause of kidney damage. Proximal tubular epithelial cells (PTECs) are highly susceptible to ischemic insults that frequently cause acute kidney injury (AKI), a potentially life-threatening condition with high mortality. Accumulating evidence has identified altered mitochondrial function as a central pathologic feature of AKI. The mitochondrial NAD^+^-dependent enzyme sirtuin 5 (SIRT5) is a key regulator of mitochondrial form and function, but its role in ischemic renal injury (IRI) is unknown. SIRT5 expression was increased in murine PTECs after IRI in vivo and in human PTECs (hPTECs) exposed to an oxygen/nutrient deprivation (OND) model of IRI in vitro. SIRT5-depletion impaired ATP production, reduced mitochondrial membrane potential, and provoked mitochondrial fragmentation in hPTECs. Moreover, *SIRT5* RNAi exacerbated OND-induced mitochondrial bioenergetic dysfunction and swelling, and increased degradation by mitophagy. These findings suggest SIRT5 is required for normal mitochondrial function in hPTECs and indicate a potentially important role for the enzyme in the regulation of mitochondrial biology in ischemia.

## Introduction

Acute kidney injury (AKI) is a life-threatening condition with high morbidity and mortality. In those who survive, outcomes vary from complete resolution to partial recovery of renal function, often with prolonged hospitalization and the longer-term risk of developing chronic kidney disease^1^. Early diagnosis and intervention are vital to limit disease progression. A leading cause of AKI is renal hypoperfusion during surgery, resulting in ischemic renal injury (IRI)^1^, suggesting that at-risk individuals would benefit from pre-emptive treatment to prevent or ameliorate AKI.

Growing evidence has suggested mitochondrial dysfunction is a major contributor to AKI^2^. Mitochondria are highly dynamic organelles required for energy production and undergo constant fission and fusion to maintain quality control^3^ and to meet metabolic requirements^4^. Mitochondrial structure and function are closely-linked: fission is required for degradation of dysfunctional mitochondria (mitophagy) and is associated with impaired energy metabolism, whereas fusion protects mitochondria from mitophagy and boosts ATP generation^3,5^. Mitochondrial dynamics are tightly regulated by nuclear-encoded GTPases (fission and fusion proteins)^6^. Fission is driven by dynamin-related protein 1 (DRP1), which on phosphorylation (S616) translocates to the mitochondrial membrane, binds to its receptors [e.g. fission 1 (FIS1) and/or mitochondrial dynamics protein 49/51 (MiD49/51)], and induces mitochondrial fragmentation^7^. Fusion is mediated by mitofusin 1/2 (MFN1/2) and optic atrophy 1 (OPA1) anchor proteins that promote fusion of the mitochondrial outer and inner membranes, respectively^6^.

Excessive mitochondrial fragmentation^8–10^ and swelling^11,12^ in renal tubules are early features of cellular injury. Recently, Perry et al. showed that maintaining mitochondrial structure following IRI in mice preserved renal function, indicating a potential therapeutic approach in AKI^13^.

The mitochondrial membrane potential (ΔΨ_M_) and the ATP pool are central determinants of mitochondrial architecture^9,14^. During ischemia, ΔΨ_M_ and ATP levels decline, driving mitochondrial fragmentation^9,14^. Notably, the ischemia-induced decline of ΔΨ_M_ has been shown to reverse F_1_F_0_-ATP synthase activity from synthesis to hydrolysis of ATP^9^. This mechanism, which is aimed at increasing the ΔΨ_M_ and preserving mitochondrial structure during hypoxia, requires a metabolic switch from oxidative phosphorylation (OXPHOS) to glycolysis to supply sufficient ATP^9^. However, renal tubular cells differ in their capacity to switch to anaerobic metabolism, resulting in large variations in the degree of injury along the nephron. The nephron segment primarily affected by ischemia is the proximal tubule (PT)^14^. Interestingly, although PT epithelial cells (PTECs) are primarily depend on OXPHOS, it has been shown that these cells can undergo a metabolic switch during ischemia, both in vivo^15^ and in vitro^16^. Therefore, boosting glycolytic ATP production in PTECs might be a therapeutic strategy to maintain mitochondrial structure and reduce ischemic injury.

The Sirtuin (SIRT) family of NAD^+^-dependent lysine (K) deacylases (KDACs) are important cellular stress sensors that regulate energy metabolism and promote mitochondrial function in organs with high metabolic demand including the kidneys^17^. Multiple lines of evidence have shown that the K-deacetylases SIRT1 and 3, play important roles in the kidney and activation during metabolic stress is thought to be a compensatory mechanism to avert mitochondrial dysfunction and protect cells from damage^18–20^. More recently SIRT5 has emerged as a central regulator of cellular energy metabolism. Unlike SIRT1 and 3, SIRT5 displays strong K-desuccinylase/demalonylase/deglutarylase activity with only very weak K-deacetylase activity^21–23^. SIRT5 primarily localizes to mitochondria, where it regulates metabolic pathways, including fatty acid oxidation (FAO) and the Krebs cycle^24,25^, but also localizes to the cytosol where it boosts glycolysis^26^. SIRT5 has been shown to enhance cardiac energy metabolism in vivo^27^ and has been implicated as a vital regulator of mitochondrial structure in mouse embryonic fibroblasts in vitro^28^*.* Zhu et al. showed that intermittent hypoxia, a treatment regimen associated with increased stress tolerance, leads to increased cardiac SIRT5 expression in rats, suggesting a protective role for SIRT5 during ischemia^29^. While this has been confirmed in the heart^24^ and brain^30^, it is unknown whether this also applies to the kidneys. Given that SIRT5 regulates both mitochondrial and glycolytic energy metabolism, and is involved in the regulation of mitochondrial structure^28^, we sought to determine the role of SIRT5 in ischemia-induced mitochondrial dysfunction in human PTECs (hPTECs) in vitro.

## Results

### SIRT5 expression increases after ischemia in PTECs in vivo and in vitro

**Figure 1:**
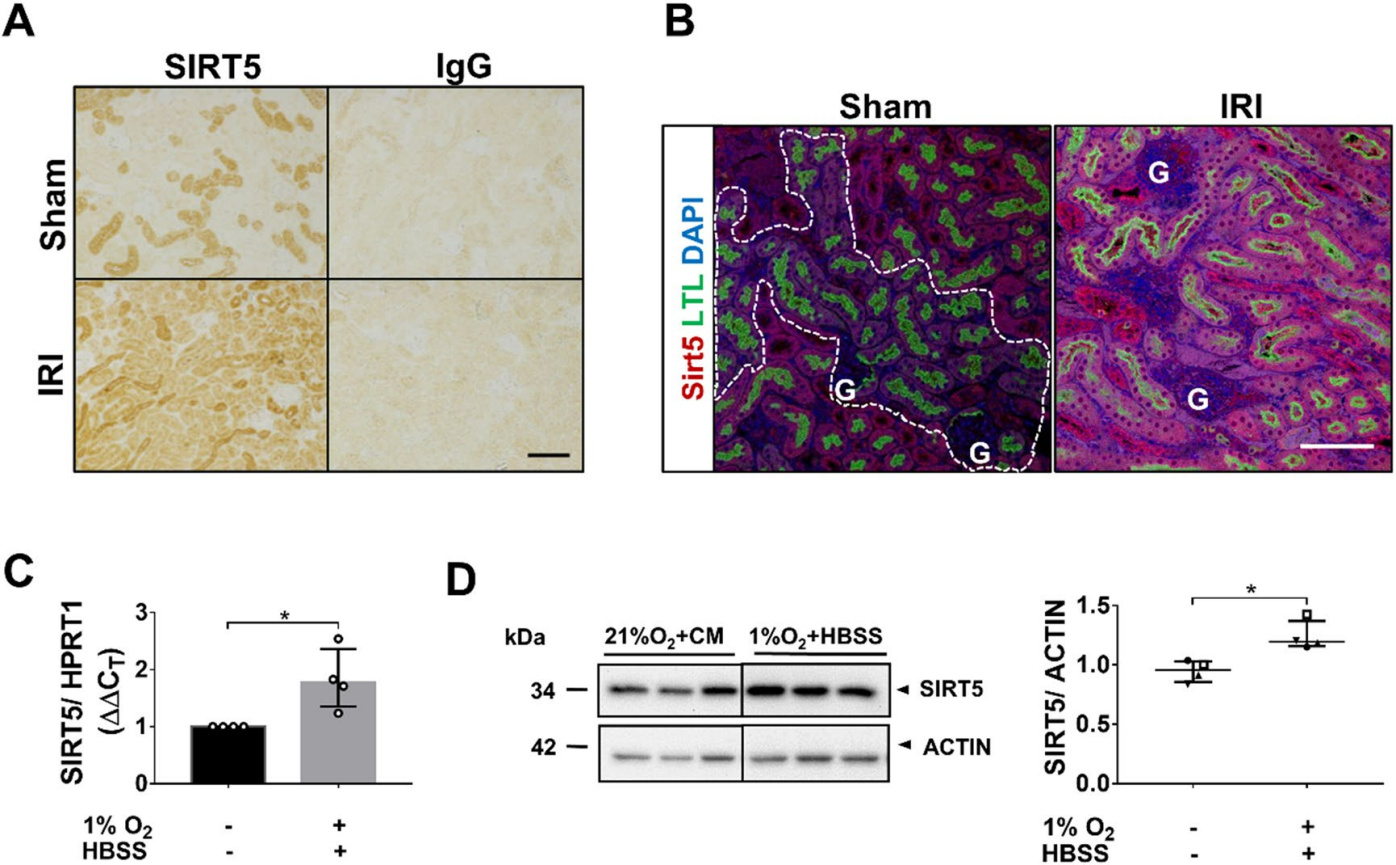
SIRT5 expression increases after ischemia in PTECs in vivo and in vitro. (**A**,**B**) Male C57BL/6J mice underwent either right uninephrectomy followed by clamping of the left renal pedicle for 30 min (IRI group; bilateral IRI) or sham surgery. Tissues were harvested 48 h post-surgery. (**A**) IHC analysis. Formalin-fixed, paraffin-embedded (FFPE) kidneys (sham and IRI) were screened for SIRT5. Rabbit IgG at an equivalent concentration served as a control. (**B**) Immunofluorescence analysis. Murine FFPE kidney sections (sham and IRI) were co-stained for SIRT5 (red) and LTL (green), a marker of PTECs. 4′,6-diamidino-2-phenylindole (DAPI) (blue) was used to visualize nuclei. Dashed lines in the sham-surgery group define low SIRT5 expression in PTs. G: Glomeruli. Scale bars: 100 µm. n = 3–5 mice/group. (**C**,**D**) hPTECs were exposed to control conditions (21%O_2_ + CM) or OND (1%O_2_ + HBSS). (**C**) Bar graph showing *SIRT5* mRNA expression. (**D**) Representative WBs and scatter plot of densitometric analysis showing SIRT5 protein levels. Whole WB shown in supplementary file (Supplementary Fig. S7a). Data are from four independent experiments with n = 3 replicates/group. mRNA levels were normalized to HPRT1. SIRT5 protein levels were normalized to actin. ImageJ was used for densitometry. To determine statistical significance of mRNA and protein data, a Mann–Whitney test was carried out. Data are median ± IQR. *p < 0.05.

Bilateral IRI increased SIRT5 protein levels in the renal cortex (Fig. 1A). Immunofluorescence co-labelling of SIRT5 with the PTEC marker, LTL, showed SIRT5 levels strongly increased in PTs (Fig. 1B). To mimic renal ischemia in vitro, an oxygen/nutrient deprivation (OND) model was developed on the premise that in vivo renal ischemia impairs both oxygen and nutrient supply, and that components in complete medium such as amino acids affect the response of tubular cells to ischemia^31,32^. Cells were exposed to both hypoxia (1% O_2_) and starvation (HBSS). Of note, OND, but not hypoxia alone, induced autophagy, a key survival pathway induced by renal ischemia in vivo^33^, highlighting the importance of combining hypoxia with nutrient deprivation to mimic the in vivo setting (Supplementary Fig. S1A,B). OND increased both SIRT5 mRNA and protein levels (Fig. 1C,D; Supplementary Fig. S1C–E).

### *SIRT5*-knockdown impairs cellular energy metabolism and induces mitochondrial fragmentation

**Figure 2:**
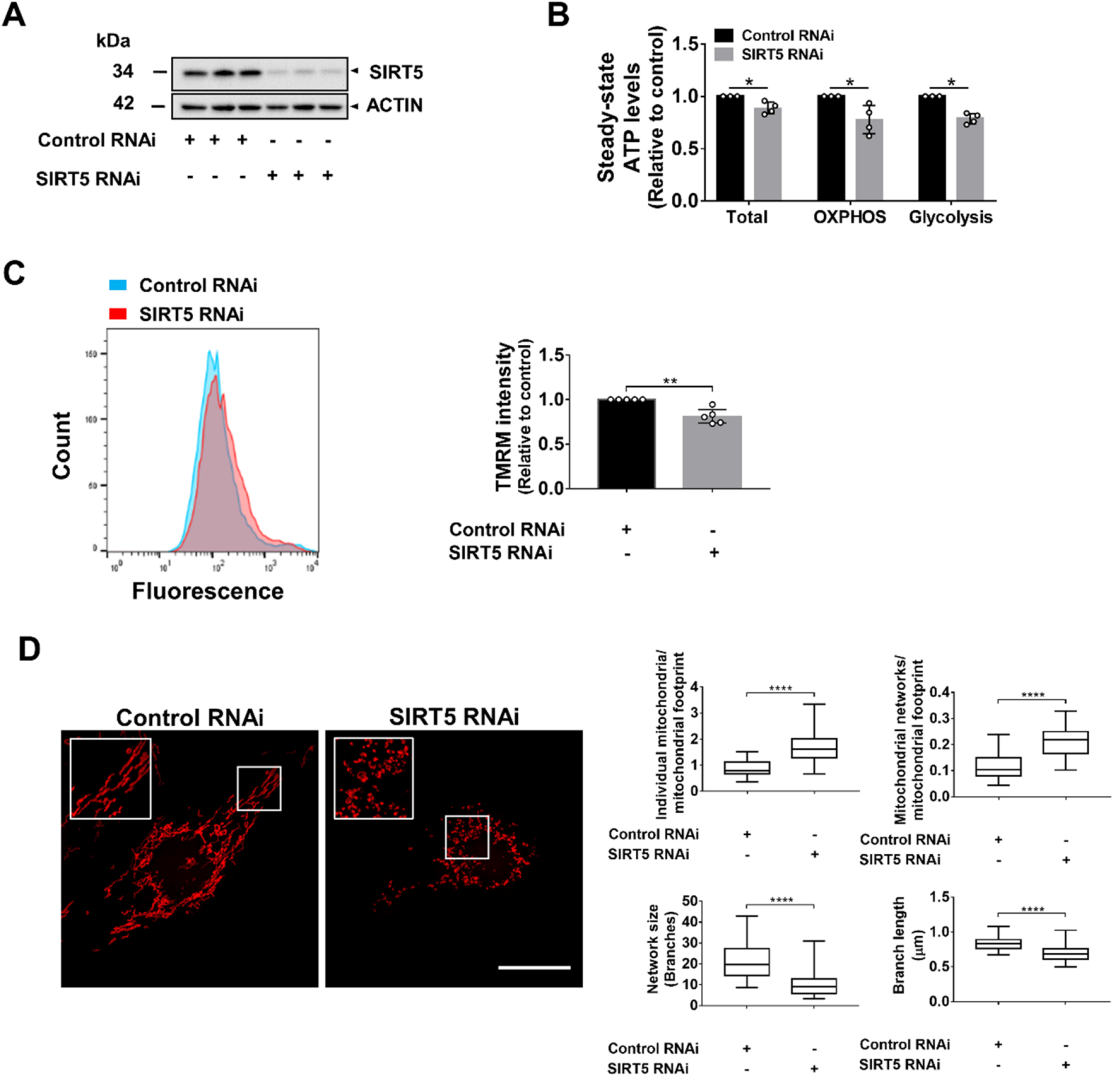
*SIRT5*-knockdown impairs cellular energy metabolism and induces mitochondrial fragmentation. *SIRT5*-knockdown by RNAi was carried out in hPTECs. Non-targeting siRNA served as control. All experiments were performed 72 h after siRNA transfection. (**A**) WB showing SIRT5 protein levels. Whole WB shown in supplementary file (Supplementary Fig. S8b). (**B**) Bar graph shows ATP levels. hPTECs (control and *SIRT5* RNAi) were analyzed without inhibitor treatment to determine total ATP levels (glycolytic + mitochondrial ATP), after treatment with 2-deoxyglucose to inhibit glycolysis (mitochondrial ATP; OXPHOS) or after treatment with oligomycin A to inhibit mitochondrial ATP synthase (glycolytic ATP; Glycolysis). ATP levels were normalized to cell number as measured by DNA quantification. Data are from four independent experiments with n = 5–6 technical replicates/group. (**C**) Bar graph showing TMRM fluorescent intensity. hPTECs (control and *SIRT5* RNAi) were incubated with TMRM (25 nM), a fluorescent dye which accumulates in mitochondria based on the ΔΨ_M_ and fluorescent intensities were determined by flow cytometry. Background fluorescence was assessed by FCCP treatment (10 µM) prior to TMRM incubation and fluorescence intensities were subtracted from the TMRM fluorescence intensities. Data are from five independent experiments with n = 2 technical replicates/group. Data are median ± IQR. To determine statistical significance a Mann–Whitney U test was applied. *p < 0.05 and **p < 0.01. (**D**) Cells were stained with Mitotracker Red CMXRos (100 nM) and imaged by confocal microscopy. Scale bar: 20 µm. (**B**) Mitochondrial morphology was quantitatively assessed using the Mitochondrial Network Analysis (MiNA) toolset (ImageJ). The number of (i) individual mitochondria and (ii) mitochondrial networks per cell were quantified and normalized to the mitochondrial footprint; (iii) mean network size (number of branches) and the mean branch length (µm) were determined. Forty-two control RNAi-treated and 51 *SIRT5* RNAi-treated cells from three independent experiments were analyzed. Box and whisker plots display median interquartile range and minimum and maximum values. Mann–Whitney U test was used to determine statistical significance. ****p < 0.0001.

*SIRT5* RNAi reduced SIRT5 protein expression by 85% (Fig. 2A). SIRT5-depletion impaired cellular energy metabolism (total ATP; Fig. 2B, Supplementary Fig. S2) but had no impact on PTEC viability (Supplementary Fig. S9). To differentiate between mitochondrial and glycolytic ATP generation, transfected cells were pre-treated with 2-deoxyglucose (2-DG) or oligomycin A (O) to inhibit glycolysis or mitochondrial metabolism, respectively. SIRT5-depletion impaired both glycolytic and mitochondrial ATP generation (Fig. 2B, Supplementary Fig. S2). ΔΨ_M_ is a fundamental parameter that regulates mitochondrial ATP synthesis^34^. To determine whether *SIRT5*-knockdown affected ΔΨ_M_ and thus contributed to the reduction in mitochondrial ATP, transfected cells were incubated with TMRM and analyzed by FACS. *SIRT5*-knockdown reduced TMRM fluorescence intensities (Fig. 2C), indicating a lower ΔΨ_M_ in *SIRT5* RNAi-treated cells. While the changes shown in Fig. 2B,C appear small, it should be stressed that the data were generated from four (ATP assay; Supplementary Fig. S2) and five (FACS analysis) independent knockdown experiments with high reproducibility. To assess whether *SIRT5*-knockdown affected mitochondrial morphology, we incubated control and *SIRT5* RNAi-treated cells with Mitotracker and assessed mitochondrial structure. SIRT5-depletion caused mitochondrial fragmentation (Fig. 2D). Semi-quantitative image analysis showed that compared with controls, *SIRT5*-knockdown increased the number of individual mitochondria/mitochondrial footprint (0.87 ± 0.28 vs 1.66 ± 0.55) and mitochondrial networks/mitochondrial footprint (0.11 ± 0.05 vs 0.21 ± 0.06), and reduced mean network size (21.1 µm vs 10.1 µm), as well as mean branch length (0.84 ± 0.1 vs 0.69 ± 0.11 µm) (Fig. 2D). These data suggest that in hPTECs, *SIRT5* RNAi impairs both glycolytic and mitochondrial ATP generation, reduces ΔΨ_M_, and stimulates mitochondrial fragmentation.

### *SIRT5*-knockdown disrupts the mitochondrial fission/fusion machinery

**Figure 3:**
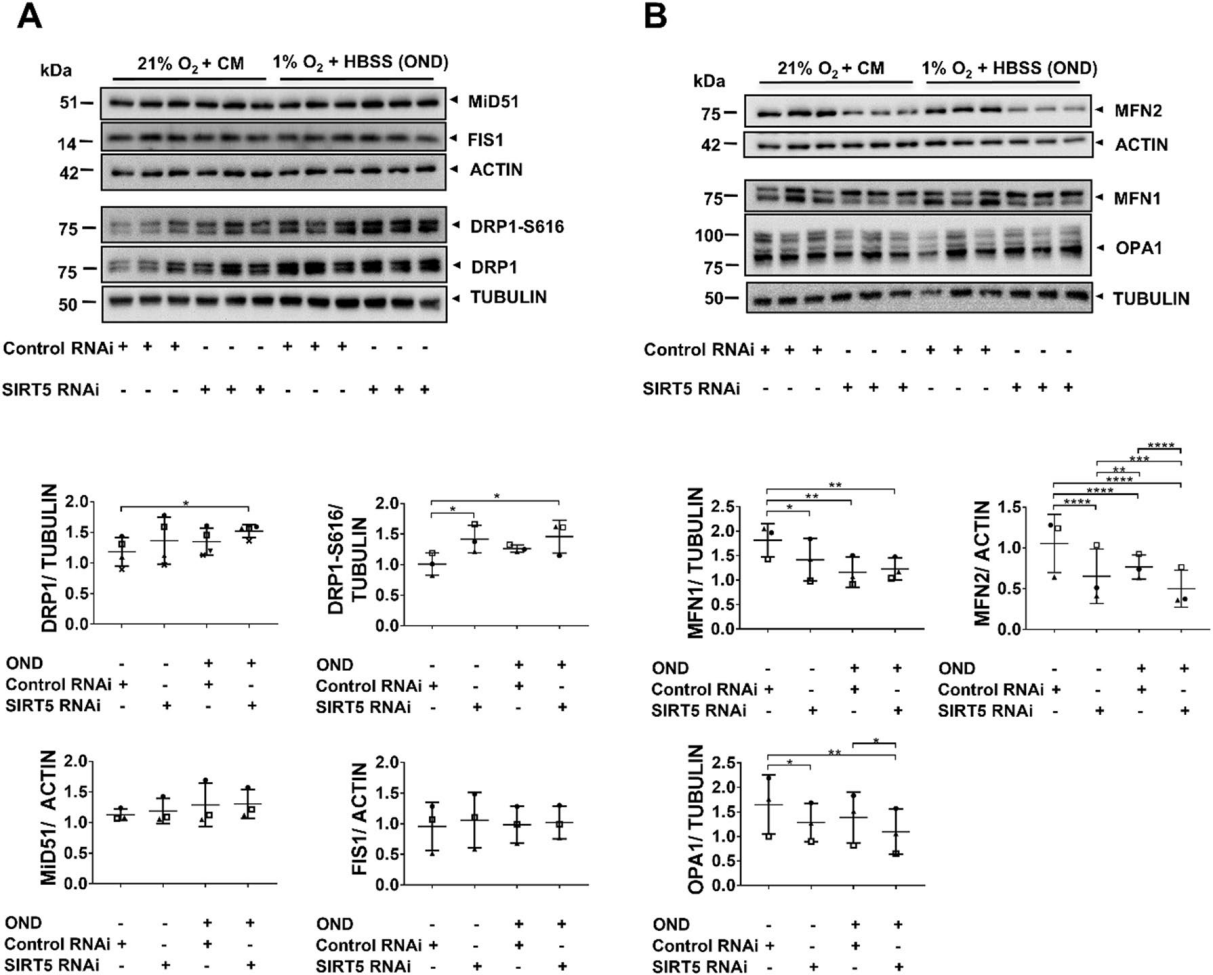
*SIRT5*-knockdown disrupts the mitochondrial fission/fusion machinery. hPTECs were transfected using siRNA against *SIRT5* or non-targeting siRNA control. Seventy-two hours post-transfection cells were exposed to 6 h of OND (1%O_2_ + HBSS) or control conditions [normoxia and complete medium (21%O_2_ + CM)]. (**A**) Representative WBs of pro-fission proteins DRP1, DRP1-S616, the DRP1 receptors mitochondrial MiD51 and FIS1. Scatter plots showing densitometry results of DRP1, DRP1-S616, MiD51 and FIS1. DRP1 and DRP1-S616 were normalized to tubulin while MiD51 and FIS1 where normalized to actin. Whole WBs shown in supplementary file (Supplementary Fig. S8c,d). (**B**) WBs of pro-fusion proteins MFN1/2 and OPA1. Scatter plots showing densitometric quantitation of MFN1, MFN2 and OPA1. Data are from three to four independent experiments with n = 3 replicates/group. MFN1 and OPA1 were normalized to tubulin while MFN2 was normalized to actin. Whole WBs shown in supplementary file (Supplementary Fig. S8e). ImageJ was used for densitometric analysis. To determine statistical significance a two-way ANOVA was carried out followed by Tukey’s post hoc test to normalize for multiple comparisons. Data are mean ± SD. *p < 0.05, **p < 0.01, ***p < 0.001 and ****p < 0.0001.

We tested whether *SIRT5*-knockdown affected the expression of mitochondrial fission/fusion proteins under control conditions, and whether this was exacerbated during OND. To assess mitochondrial fission, we screened for DRP1, DRP1-S616 and MiD51 and FIS1. Compared with control RNAi-treated cells, DRP1 levels were increased in SIRT5-depleted cells exposed to OND (Fig. 3A). DRP1-S616 levels can be normalized to total DRP1 protein levels and represented as DRP1-S616/ DRP1 ratio, if total DPR1 protein are not affected by the treatment (i.e. RNAi or OND) as this masks biologically-relevant changes in DRP1-S616 (Supplementary Fig. S3). Given that, in the present study, total DRP1 levels increased with the treatment (Fig. 3A), we decided to normalize DRP1-S616 to tubulin to better represent the underlying mechanism. As shown in Fig. 3A, SIRT5-depletion alone increased DPR1-S616 phosphorylation compared with control RNAi. This increase was independent of OND, since DRP1-S616 levels did not change in control RNAi-treated cells exposed to OND, suggesting that SIRT5-depletion affected DRP1-S616 phosphorylation to promote fission^35^. Interestingly, neither *SIRT5* RNAi nor OND affected levels of the DRP1 receptors, MiD51 and FIS1 (Fig. 3A). Next, we measured expression of the three key pro-fusion proteins MFN1, MFN2 and OPA1 (Fig. 3B). Under control conditions, *SIRT5*-knockdown reduced MFN1, MFN2 and OPA1 relative to control RNAi, indicating that SIRT5 prevents fragmentation by preserving the pro-fusion system. OND further decreased OPA1 and MFN2, leading to significant differences between control RNAi- and *SIRT5* RNAi-treated cells (Fig. 3B). Notably, OND-treatment also reduced MFN1 and MFN2 levels in control RNAi-treated cells, but did not significantly decrease total OPA1 levels. While the WBs shown in Fig. 3A,B are representative blots from one SIRT5-knockdown experiment, it should be emphasized that the data shown in the scatter plots are pooled from 3–4 independent SIRT5-knockdown experiments (biological replicates), which were all carried out in triplicate (technical replicates). Taken together, these data indicate that *SIRT5* RNAi causes mitochondrial fragmentation by affecting central components of the mitochondrial fission/fusion machinery.

### *SIRT5*-knockdown affects l-OPA1 processing by OMA1 and YME1L

**Figure 4:**
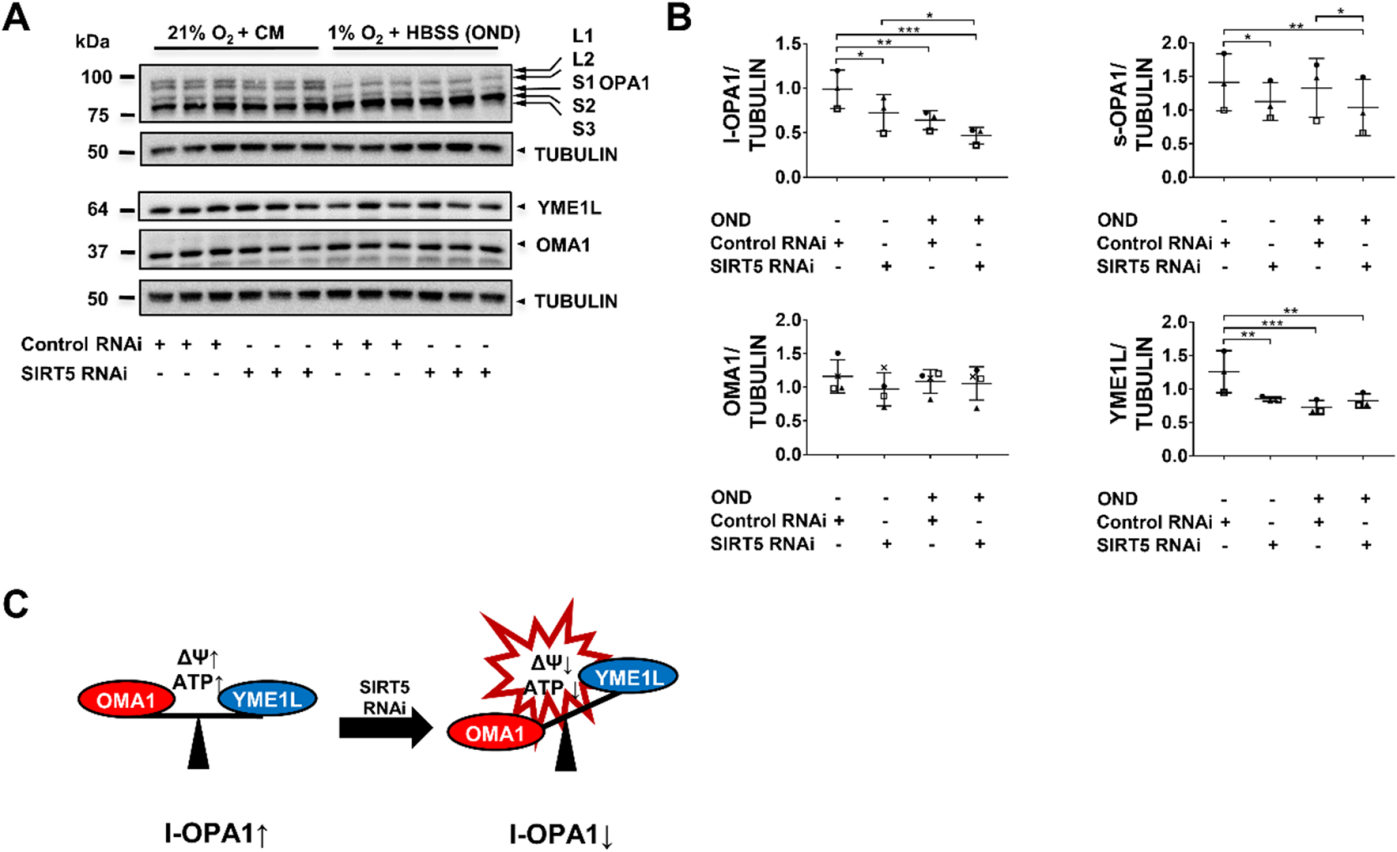
*SIRT5*-knockdown affects OPA1 processing by OMA1 and YME1L. hPTECs were transfected with siRNA against *SIRT5* or non-targeting siRNA control. Seventy-two hours post-transfection cells were exposed to 6 h OND (1%O_2_ + HBSS) or normoxia and complete medium (21%O_2_ + CM). (**A**) WBs showing protein levels of OPA1 (five isoforms: L1, L2, S1–3) and the OPA1 processing proteases, OMA1 (ATP-independent) and YME1L (ATP-dependent). Whole WBs shown in supplementary file (Supplementary Fig. S8f) (**B**) Scatter plots showing densitometric quantitation of long (l)-OPA1, short (s)-OPA1, OMA1 and YME1L. Data are from three to four independent experiments with n = 3 replicates/group. Tubulin was used as loading controls. ImageJ was used for densitometric analysis. To determine statistical significance a two-way ANOVA was carried out followed by Tukey’s post hoc test to normalize for multiple comparisons. Data are mean ± SD. *p < 0.05, **p < 0.01, and ***p < 0.001. (**C**) Schematic of the proposed model of *SIRT5* RNAi-induced l-OPA1 processing by OMA1 and YME1L. In control RNAi-transfected cells, l-OPA1 processing is limited due to the inhibitory effect of YME1L on OMA1. *SIRT5* RNAi induced OMA1-mediated degradation of YME1L resulting in increased l-OPA1 processing.

The pro-fusion protein OPA1exists as long (l)-OPA1 (L1/2) and short (s)-OPA1 (S1–3) isoforms that are key to mitochondrial membrane fusion^36–38^ and cristae formation^39^, respectively. The peptidases OMA1 (ATP-independent) and YME1L (ATP-dependent), which undergo reciprocal degradation, both convert l-OPA1 to s-OPA1^36,40^. However, only OMA1 can transform all l-OPA1 to s-OPA1, thereby promoting fission^36^, because all l-OPA1 isoforms have a OMA1 cleavage site (S1), while only ~ 50% have a YME1L cleavage site (S2)^40^. In healthy mitochondria, high ATP levels activate YME1L and degradation of OMA1 to promote fusion^36^. Decline in ATP levels and ΔΨ_M_ impair YME1L activity and activate OMA1, which degrades YME1L to promote fission^36^. We examined whether SIRT5*-*depletion altered l-OPA1 processing in hPTECs (Fig. 4A). *SIRT5*-knockdown significantly reduced l-OPA1 protein levels under normoxia which was exacerbated by OND (Fig. 4B), suggesting that SIRT5 is involved in l-OPA1 proteolysis. Notably, l-OPA1 levels also declined in OND-treated control RNAi cells. This is consistent with the finding that ischemia drives l-OPA1 degradation (due to OMA1-mediated YME1L proteolysis)^41^. Next, we assessed whether OMA1 may drive l-OPA1 proteolysis (Fig. 4B). Both *SIRT5* RNAi and OND reduced YME1L protein, while OMA1 levels remained unchanged, suggesting that OMA1-mediated YME1L degradation may be the cause of the increased l-OPA1 proteolysis (Fig. 4C). Proteolytic cleavage of l-OPA1 provokes a shift to s-OPA1^38^, which has been shown to promote mitochondrial bioenergetics and cristae formation^39^. Intriguingly, s-OPA1 levels declined in SIRT5-depleted cells (in normoxia and OND) and were unaffected in the control RNAi-treated OND group (Fig. 4B), suggesting *SIRT5 RNAi* may also influence s-OPA1 degradation. Taken together, these data indicate that *SIRT5* RNAi affects OPA1 proteolysis, possibly through a YME1L/OMA1-dependent mechanism (Fig. 4C).

### SIRT5-depletion exacerbates OND-induced bioenergetic dysfunction

**Figure 5:**
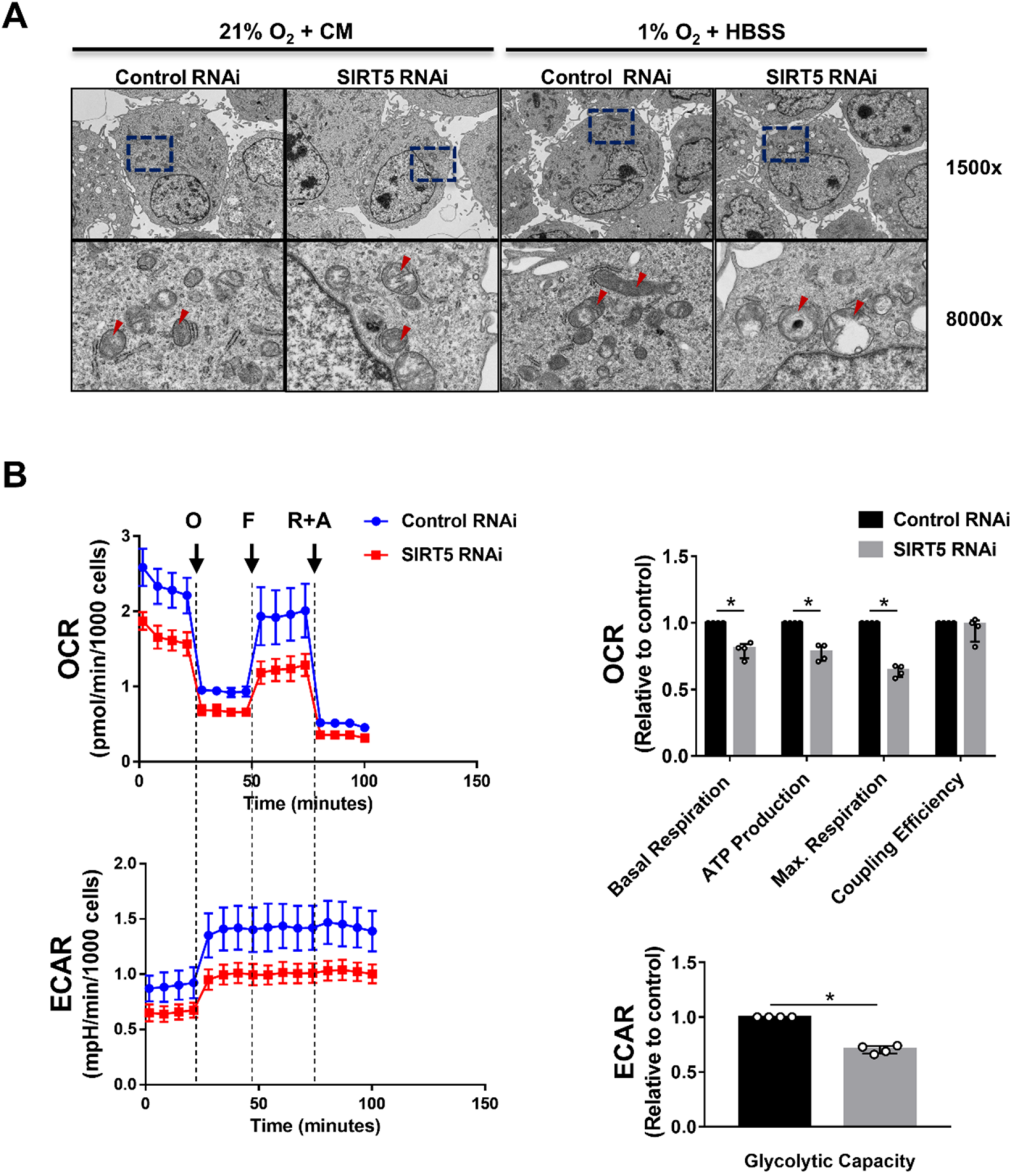
SIRT5-depletion exacerbates OND-induced bioenergetic dysfunction. hPTECs were transfected with siRNA against *SIRT5* or non-targeting siRNA control. Seventy-two hours post-transfection cells were exposed to 6 h of oxygen and nutrient deprivation (OND) (1%O_2_ + HBSS) or normoxia and complete medium (21%O_2_ + CM). (**A**) TEM images showing mitochondria (red arrows). Magnification: ×1500 and ×8000. Lower panels show higher magnification of the area indicated by blue squares. (**B**) Summary of Seahorse XFp Mito Stress Test assay of cells exposed to 6 h OND. Representative profile of Mito Stress Test data showing oxygen consumption rate (OCR) and extracellular acidification rate (ECAR) with arrows indicating injection of the specific stressors oligomycin (O), FCCP (F) and Rotenone plus antimycin A (R + A). Relative values of the parameters normalized to the non-targeting siRNA control. Data are from four independent experiments with n = 3 technical replicates/group. Wave software (Agilent) was used for data analyses. Mann–Whitney U test was used to determine statistical significance. Data are median ± IQR. *p < 0.05.

Mitochondrial swelling and the resulting metabolic dysfunction are central pathophysiologic features in ischemia-induced PTEC injury^42–46^. Next, we analyzed the impact of *SIRT5*-knockdown on mitochondrial ultrastructure. *SIRT5* RNAi induced mitochondrial swelling during normoxia, which was amplified by OND (Fig. 5A), suggesting that SIRT5 prevents OND-induced changes in mitochondrial ultrastructure. This finding was confirmed by quantitative image analysis (Supplementary Fig. S4). Subsequently, we assessed the effect of *SIRT5*-knockdown on mitochondrial function after OND. *SIRT5* RNAi significantly impaired mitochondrial and glycolytic energy metabolism, evident as a reduction in basal respiration, respiration coupled to ATP production, maximal respiration and glycolytic capacity (Fig. 5B). These data suggest that *SIRT5*-knockdown exacerbates OND-induced mitochondrial dysfunction.

### SIRT5-depletion enhances the OND-induced reduction in mitochondrial mass

**Figure 6:**
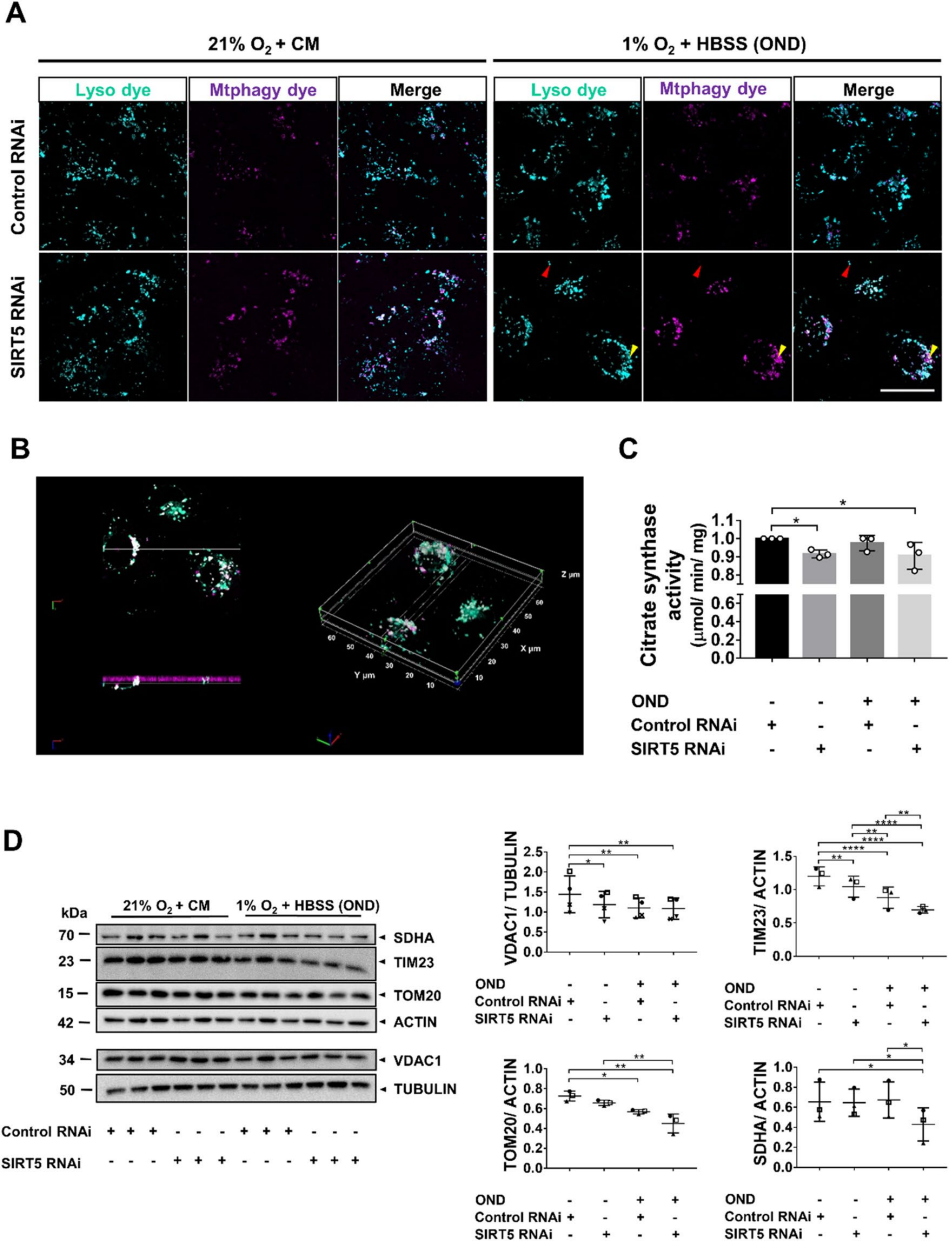
SIRT5-depletion aggravates OND-induced mitochondrial mass reduction. hPTECs were transfected using siRNA against *SIRT5* or non-targeting siRNA control. Seventy-two hours post-transfection cells were exposed to 6 h of OND (1%O_2_ + HBSS) or normoxia and complete medium (21%O_2_ + CM). (**A**) Maximum projection laser-scanning confocal micrographs showing lysosomes (cyan; red arrow) and lysosome-engulfed mitochondria (magenta; yellow arrow). hPTECs were incubated with a fluorescent, pH-dependent probe (Mtphagy dye; magenta), exposed to 21%O_2_ + CM or OND and subsequently, incubated with a lysosomal dye (Lyso dye; cyan). Scale bar: 25 µm. (**B**) Representative 3D image of engulfed mitochondria (magenta) in lysosomes (cyan). Co-localized areas appear white. Z-stacks were analyzed using Image-Pro Premier 10 (MEDIA CYBERNETICS). (**C**) Bar graph showing citrate synthase (CS) activity as a measure of mitochondrial mass. CS activity/sample was normalized to total protein content. Data are from three independent experiments with n = 3 technical replicates/group. To determine statistical significance a two-way ANOVA was carried out followed by Tukey’s post hoc test to normalize for multiple comparisons. Data are mean ± SD. *p < 0.05. (**D**) WB showing protein levels of SDHA, TIM23, TOM20 and VDAC1. Scatter plots showing the densitometric analysis for SDHA, VDAC1, Tim23 and TOM20. VDAC1 was normalized to tubulin and SDHA, TIM23 and TOM20 were normalized to actin. Whole WBs shown in supplementary file (Supplementary Fig. S8g) ImageJ was used for densitometric analysis. Data are from three to four independent experiments with n = 3 technical replicates/group. To determine statistical significance a two-way ANOVA was carried out followed by Tukey’s post hoc test to normalize for multiple comparisons. Data are mean ± SD. *p < 0.05, **p < 0.01, ***p < 0.001 and ****p < 0.0001.

Mitochondrial fragmentation and mitochondrial dysfunction are necessary for autophagy-mediated degradation (mitophagy)^47^. Next, we assessed whether *SIRT5* RNAi increased mitophagy during normoxia and/or OND using a pH-dependent, fluorescent probe (Mtphagy Dye) and a lysosomal dye (Lyso Dye). SIRT5-depletion increased mitophagy during normoxia, which was further increased by OND compared with control RNAi-treated cells (Fig. 6A, Supplementary Fig. S5). Co-localization of the Mtphagy and Lyso Dyes was confirmed by 3D image analysis (Fig. 6B). To determine whether *SIRT5*-knockdown affected mitochondrial mass under normoxia or OND, we conducted a citrate synthase (CS) activity assay^48^, quantified mitochondrial marker proteins and determined mitochondrial DNA (mtDNA)/nuclear DNA (nDNA)-ratios (Fig. 6C,D and Supplementary Fig. S6). *SIRT5* RNAi reduced CS activity under both normoxia and OND compared with control RNAi-treated cells. We subsequently screened RNAi-treated cells for the mitochondrial markers: succinate dehydrogenase A (SDHA), voltage-dependent anion channel 1 (VDAC1), translocase of inner membrane 23 (TIM23), and translocase of outer membrane 20 (TOM20). Under normoxia, *SIRT5* RNAi significantly decreased VDAC1 and TIM23 protein levels compared with control RNAi, suggesting that *SIRT5*-knockdown reduces mitochondrial mass (Fig. 6D). This was exacerbated by OND as TIM23, TOM20 and SDHA protein levels declined further in the *SIRT5* RNAi-treated cells, resulting in significant differences between control RNAi- and *SIRT5* RNAi-treated cells (Fig. 6D). Notably, SIRT5-depletion caused an increase in mtDNA/nDNA-ratio, perhaps as a compensatory response to bioenergetic dysfunction, which then decreased after OND (Supplementary Fig. S6). These data suggest that *SIRT5* RNAi increases mitochondrial degradation in OND.

## Discussion

Renal ischemia leading to IRI is a major cause of AKI^1^. The renal cell type primarily affected by IRI is PTECs due to their high metabolic turnover, reliance on mitochondrial function and limited ability to switch to anaerobic metabolism under low-oxygen conditions^9,14^. Under ischemic conditions, PTECs enter an ATP crisis within minutes, resulting in tubular cell death and tissue damage^14,49^. Mitochondrial dysfunction has emerged as a major contributor to ischemic damage in AKI^2^. In particular, excessive mitochondrial fragmentation and swelling have been shown to be important pathological features of IRI that impair mitochondrial function and may drive disease progression^11,14,42–46,50^. Conversely, preserving mitochondrial structure can reduce IRI^9,13^. Mitochondria contribute to IRI by aggravating ATP-depletion during ischemia and increasing ROS production during reperfusion^24,51–55^. However, recent findings on ATP levels in the renal tubule following ischemia, while confirming increased susceptibility of the proximal tubule to ATP depletion, also noted that incomplete or delayed ATP recovery correlated with more fibrosis^56^. This suggests that tubular damage might be limited by (A) preserving mitochondrial structure and (B) some initial dampening of mitochondrial function and ROS production and allow more rapid recovery of ATP generation. PTECs must regain mitochondrial function post-injury, since failure to do so will exacerbate tubular damage^15,44^. The de-succinylase/-manolynase/-glutarylase SIRT5 has been shown to regulate both mitochondrial^24,25^ and glycolytic^26^ energy metabolism, as well as mitochondrial structure^28^. However, its role in the kidney and in PTECs, remains largely unknown. In this study, we showed that SIRT5 is an ischemia-inducible enzyme in PTECs both in vivo and in vitro. Furthermore, in this cell type SIRT5-depletion impaired both glycolytic and mitochondrial bioenergetics and provoked mitochondrial fragmentation. Finally, we demonstrated that *SIRT5* RNAi increased OND-induced mitochondrial swelling, reduced mitochondrial function and enhanced mitophagy in hPTECs in vitro, suggesting that SIRT5-depletion overall reduces mitochondrial function in this model.

To more accurately mimic renal ischemia in vitro and because components in complete medium such as amino acids affect the response of tubular cells to ischemia^31,32^, we developed an OND model, which combines oxygen and nutrient deprivation. Exposure of hPTECs to OND revealed that SIRT5 expression is regulated by ischemia, a finding that is consistent with our observation in a mouse IRI model. In addition, we showed hypoxia increases SIRT5 expression in vitro. This finding aligns with a study in rats in which intermittent hypoxia, a treatment regimen known to provide cardioprotection, increased cardiac SIRT5 levels^29^, suggesting that inducing SIRT5 in hPTECs may be a compensatory mechanism to protect cells from ischemic injury. Although the OND model mimics some aspects of IRI (hypoxia and nutrient depletion) there are some limitations to the model, since nutrient deprivation may reduce the accumulation of Krebs cycle intermediates, which are important drivers of IRI in vivo^54^.

Mitochondrial fragmentation and swelling have been shown to be pathologic features of AKI^9,11,12,42,43^. Conversely, preventing mitochondrial fragmentation in vivo can reduce IRI^9^ and improve recovery^13^, suggesting that targeting mitochondrial dynamics may be a therapeutic strategy in AKI. We have demonstrated that in hPTECs in vitro SIRT5-depletion induces mitochondrial fragmentation by modulating the mitochondrial fission (DRP1-S616) and fusion (MFN1/2 and OPA1) machinery and impairs metabolic homeostasis during normoxia and OND. In keeping with our findings, Li et al.^57^ recently showed that SIRT5-depleted HK-2 cells display fragmented mitochondria. While these authors also showed that *SIRT5* RNAi increased fission via DRP1-S616, they did not investigate the effect of SIRT5-depletion on pro-fusion factors. Notably, we found that OND did not further increase DRP1-S616 phosphorylation in *SIRT5* RNAi-treated cells, but did cause a further decline in MFN2 and l-OPA1 levels (Fig. 3). This observation suggested that SIRT5-depletion per se may have saturated this pro-fission response, while pro-fusion mechanisms were only partly affected, maintaining a degree of mitochondrial inner^39^ and outer^58^ membrane fusion competency in normoxia. Given that mitochondrial fission facilitates mitochondrial quality control^3^, it is tempting to speculate that on one hand, increased fission may enhance selective degradation of dysfunctional mitochondria, while on the other, functional mitochondria may maintain fusion competency, to enhance overall bioenergetic output.

Mitochondrial architecture is determined by two tightly-linked elements, the ΔΨ_M_ and the ATP pool, and their depletion in ischemia, is a fundamental driver of mitochondrial fragmentation^14^ and swelling^59^. Rainbolt et al.^36^ showed that in human neuroblasts the combination of ΔΨ_M_ loss and ATP-depletion caused mitochondrial fragmentation through increased l-OPA1 degradation due to an imbalance between the l-OPA1 processing enzymes, OMA1 and YME1L. In hPTECs, SIRT5-depletion increased l-OPA1 processing, possibly by disrupting the OMA1/YME1L equilibrium (Fig. 4C). The fact that SIRT5-depletion reduced both ΔΨ_M_ and ATP levels, which activates OMA1 and reduces YME1L activity^36^, respectively, suggests that mitochondrial fragmentation in hPTECs may be a consequence of metabolic disturbance. However, measuring OMA1 and YME1L protein levels is only an indication of their enzyme activities since they undergo reciprocal proteolysis^36^. While it is generally accepted that SIRT5 boosts enzymes involved in glycolytic and mitochondrial energy metabolism^26,60^, it remains to be tested whether the effect of *SIRT5* RNAi on mitochondrial structure is a consequence of decreased bioenergetics or is more direct through regulation of fission/fusion proteins. As mentioned earlier, mitochondrial swelling in PTECs is a hallmark of ischemic AKI^11,50^. Here, we showed that SIRT5-depletion caused mitochondrial swelling in PTECs, which was enhanced by OND (Fig. 5 and Supplementary Fig. S4). Interestingly, studies in the ischemic canine heart found that mitochondrial swelling correlates with ATP-depletion^61^ and also that increased mitochondrial matrix volume stimulated respiration rates^59^, suggesting that swelling might be a compensatory mechanism to adapt to energetic stress. The observation that SIRT5-depletion impaired cellular bioenergetics suggests that a minor reduction in ATP levels may be the underlying cause, leading to more pronounced OND-induced swelling in hPTECs.

Mitochondrial form and function are tightly-linked and excessive fragmentation is associated with reduced bioenergetic output^13^, as well as increased susceptibility to mitophagy^3^. Our in vitro data showed that SIRT5-depletion decreased bioenergetic output and increased mitophagy, leading to mitochondrial mass decline after OND (Fig. 6). These data therefore hinted that *Sirt5* deletion would exacerbate IRI. Notably, a recent study by Chiba et al.^62^ showed that *Sirt5*^*−/−*^ mice are protected from IRI. The group revealed that SIRT5 ablation reduces mitochondrial function (i.e. complex II activity) as well as stimulates a switch from mitochondrial to peroxisomal FAO and proposed that these may be the underlying protective mechanisms, possibly by reducing oxidative stress. The former mechanism is in line with a variety of studies, which revealed that reducing mitochondrial function by inhibition of the ETC complexes I^51–53,63^, II^24,54^ or F_1_F_0_-ATP synthase (complex V)^64^, alleviated ischemic injury. The latter mechanism, however, presupposes that the cell type protected contains peroxisomes. Studies in mice, rats and humans have shown that only the PT segments S2 (in the cortex) and S3 (in the outer medulla) have peroxisomes, while cortical PT S1 segments appear to lack this organelle^65–68^. These data, together with the observation that SIRT5 regulates the activities of complex I, II and V in renal homogenates^62^, renal mitochondrial extracts (Supplementary Fig. S8), hepatic homogenates^69^ and cardiac mitochondria^70^, suggest that some reduction in mitochondrial function might be an additional mechanism for amelioration of IRI in *Sirt5*^*−/−*^ kidneys.

In summary, the present study showed that SIRT5 is an ischemia-inducible enzyme that regulates mitochondrial form/function in hPTECs. Based on our findings in hPTECs, we propose that SIRT5 is a key enzyme that facilitates metabolic homeostasis, protects mitochondria from fragmentation/degradation, and decreases susceptibility to OND-induced mitochondrial dysfunction. Nevertheless, it should be stressed that further studies will be needed to provide a comprehensive understanding of the function of SIRT5 in the ischemic kidney in vivo, particularly in the light of the findings of Chiba et al.^62^. Given that mitochondrial dysfunction and, perhaps more importantly, recovery of mitochondrial function are major determinants of disease progression, especially since they may determine the transition from acute to chronic kidney disease, it will be important to analyse the role of SIRT5 in a more chronic setting.

## Materials and methods

### Chemicals

Unless otherwise indicated, all chemicals were from SIGMA-ALDRICH.

### Mouse breeding and maintenance

All experimental procedures were carried out in compliance with the Animal Research: Reporting of In Vivo Experiments (ARRIVE) guidelines, approved by the UCL Animal Welfare and Ethics Review Board (Royal Free Campus) and performed under UK Home Office Project license (Project licence P6377F606). Male C57BL/6J mice were bred in-house and housed in specific pathogen-free conditions with a diurnal 12 h light/dark cycle and free access to water and standard chow. Male mice age 8–10 weeks were used for experiments.

### Ischemic renal injury (IRI)

#### Bilateral ischemia

Mice underwent IRI or sham surgery (n = 3–5/group) as described previously^71^. Briefly, mice were anesthetized with 2% isoflurane, injected with Carprofen (5 mg/kg; subcutaneous) as an analgesic, placed on a heating pad (37 °C) and underwent a midline laparotomy. For IRI, the right kidney was removed and the left renal pedicle clamped for 30 min using a vascular clamp (S&T, B-1 V). The clamp was removed, the abdomen closed, the wound sanitized with Videne Solution and 1 ml warm (37 °C) isotonic saline injected subcutaneously. Sham-operated animals were subjected to the same procedure, but without removal of the right kidney and clamping of the left renal pedicle. Kidneys were harvested 48 h post-surgery, fixed in 4% formaldehyde in phosphate-buffered saline (PBS), paraffin-embedded and sectioned.

### Cell culture and in vitro ischemia

The human proximal tubular epithelial cell line HKC-8^72–74^ was used. All cell culture experiments were performed at least three times in triplicate. Cells were grown in complete medium [CM: DMEM/F12 (gibco) supplemented with 5% fetal bovine serum (FBS, seralab)], and cultured at 37 °C in a humidified atmosphere of 5%CO_2_. To mimic ischemia, cells were seeded into 6-well plates (Corning) and incubated in either CM or HBSS supplemented with 10 mM HEPES (nutrient deprivation) under either normoxia (21%O_2_) or hypoxia (1%O_2_; BOC) for 6–24 h at 37 °C. Cultures were tested for mycoplasma contamination (LookOut Mycoplasma PCR Detection Kit, SIGMA-ALDRICH) and were negative. For details see Supplementary Methods.

### *SIRT5* siRNA knockdown

hPTECs were transfected with either the ON-TARGETplus *SIRT5* siRNA pool or control siRNA pool (Dharmacon) using Dharmafect-1 (Dharmacon), according to the manufacturer’s protocol. Cells were transfected in bulk (T25 flasks) and plated for experiments 48 h post-transfection. Knockdown efficiency was determined by Western blotting (WB) 72 h post-transfection. For details see Supplementary Methods.

### Assessment mitochondrial membrane potential (ΔΨ_M_) by FACS

Transfected (control and *SIRT5* RNAi) hPTECs were trypsinised, pelleted, resuspended in HEPES-buffered, phenol red-free DMEM supplemented with 1 mM sodium pyruvate (FACS medium), diluted to 4 × 10^5^ cells/ml and aliquoted into 2 tubes/sample (1 ml/tube). To assess background fluorescence intensities, 10 µM carbonyl cyanide-4-(trifluoromethoxy)phenylhydrazone (FCCP) was added and incubated at 37 °C for 20 min. Tetramethylrhodamine, methyl ester (TMRM) (25 nM final concentration) was added to all tubes and incubated at 37 °C for 30 min. Fluorescence intensities were measured using a Moxi GO cytometer (ORFLO) and data analyzed using FLOWJO (BD).

### Assessment of mitochondrial morphology

To assess mitochondrial morphology, transfected (control and *SIRT5* RNAi) hPTECs were incubated with Mitotracker Red CMXRos in CM for 45 min at 37 °C and fixed with 4% formaldehyde (Thermo Fisher Scientific) in PBS. Nuclei were stained with DAPI (0.1 µg/ml), cells were mounted (FluorSave, MERCK) and imaged on a TCS SP8 confocal microscope (Leica). The Mitochondrial Network Analysis toolset (Valente et al.^75^) for ImageJ was used to assess mitochondrial morphology; 42 control and 51 *SIRT5* RNAi-treated cells from three independent experiments were analyzed.

### ATP quantification

To assess mitochondrial and glycolytic ATP generation, transfected (control and *SIRT5* RNAi) hPTECs were either incubated with 25 mM 2-Deoxy-d-glucose in glucose-free DMEM supplemented with 5% FBS for 1 h or 2 µM oligomycin A in glucose-containing DMEM supplemented with 5% FBS for 25 min, respectively. The ATPlite Assay (Perkin Elmer) was performed according to the manufacturer’s protocol. ATP levels were normalized to cell number (CyQUANT; Life Technologies). Luminescence (ATP) and fluorescence (DNA) were measured with a Mithras LB 940 Multimode plate-reader (BERTHOLD).

### Ultrastructural analysis of mitochondria

For TEM, transfected hPTECs (control and *SIRT5* RNAi) were exposed to 6 h OND or control conditions, trypsinised, centrifuged, fixed in 2.5% glutaraldehyde buffered with 100 mM sodium cacodylate (pH 7.2; agar scientific) and embedded in Agar 100 epoxy resin. Ultrathin sections (90 nm) were cut, collected on a copper grid, stained and examined on a JEOL 1400 microscope with an AMT XR80 digital camera. For details see Supplementary Methods.

### Assessment of mitochondrial function by respirometry

Oxygen consumption rate (OCR; a measure of mitochondrial function) and extracellular acidification rate (ECAR; a measure of glycolysis) were assessed in transfected (control and *SIRT5* RNAi) hPTECs after 6 h OND using a Seahorse XFp Analyzer (Agilent). After assessment of basal respiration, oligomycin (2 μM), FCCP (0.75 μM) and rotenone (1 μM) with antimycin A (1 μM) were sequentially injected. For each condition, measurements of OCR and ECAR were carried out in quadruplicate, 3 min apart. OCR and ECAR were normalized to total cell number/well (CyQUANT, Life Technologies). Data were analyzed using the Wave software (Agilent). For details see Supplementary Methods.

### Immunohistochemistry

Immunohistochemistry (IHC) was performed on formalin-fixed paraffin-embedded (FFPE) sections (3 μm) after antigen-retrieval (1 mM EDTA pH8; 100 °C; 10 min in a microwave at full power) followed by blocking with 10% normal goat serum (NGS), 1% bovine serum albumin (BSA) in Tris-buffered saline (TBS) for 1 h. Sections were incubated overnight at 4 °C with anti-SIRT5 antibody (D8C3; Cell Signaling) or non-specific, control rabbit IgG (R&D Systems) at an equivalent concentration, diluted in 5% NGS in TBS. Endogenous peroxidase activity was blocked using aqueous 0.3% H_2_O_2_ (15 min). For immunodetection, Dako EnVision + /HRP reagent was applied according to the manufacturer’s protocol. Sections were mounted with mounting medium (Dako). Images were collected on a Nikon ECLIPSE Ci-L microscope with a Digital Sight D5-Fi2 camera and analyzed using ImageJ (NIH).

### Immunofluorescence

Antigen-retrieval and blocking were as described for IHC. Sections were incubated with fresh 50 mM NH_4_Cl (15 min) to quench free aldehyde groups and blocked in 10% NGS, 1% BSA in TBS (1 h). Sections were incubated with either fluorescein-labelled Lotus Tetragonolobus Lectin (LTL; VECTOR LABORATORIES), a proximal tubular cell marker, and anti-SIRT5 antibody (Cell Signaling, 8782), or control rabbit IgG (R&D Systems) overnight at 4 °C followed by incubation for 1 h at room temperature with a secondary Alexa 647-labelled antibody (abcam). Nuclei were visualized with DAPI. Sections were mounted with FluorSave (Millipore), imaged on a TCS SP8 confocal microscope (Leica) and analyzed with ImageJ (NIH).

### Gene expression analysis

Total RNA was extracted using the RNeasy Mini kit (QIAGEN) according to the manufacturer’s instructions. RNA concentration was quantified with a NanoDrop 8000 (Labtech) spectrophotometer. RNA (2 µg) was reverse transcribed (High-Capacity cDNA Kit) and qPCR (LightCycler 96, Roche) performed using the 2xqPCRBIO SyGreen (PCR BIOSYSTEMS) reaction mix. Primer sequences are shown in Supplementary Table 1. qPCR conditions were: initial denaturation and polymerase activation at 95 °C (10 min) followed by 40 cycles of: 95 °C (10 s); 60 °C (10 s); and 72 °C (10 s). Melting curve analysis was performed using a cycle of 95 °C (10 s); 65 °C (60 s); and 97 °C (1 s). Hypoxanthine phosphoribosyltransferase 1 (HPRT1) served as an internal control for human studies. Gene expression was determined by ΔΔCt.

### Western blot analysis

Cells and tissue were lysed in RIPA buffer supplemented with Halt Protease and Phosphatase Inhibitor Cocktail (Thermo Fisher Scientific) and pepstatin A. Protein concentration was determined (DC Assay; BIO-RAD), with BSA as a standard. Samples (10-25 µg) were denatured in 4 × Laemmli buffer (BIO-RAD) supplemented with β-mercaptoethanol (0.71 M), separated on 8 or 12% Tris-based polyacrylamide gels in Tris–glycine buffer [5 mM Tris, 50 mM glycine, 0.02% (w/v) SDS, pH 8.3] and transferred to PVDF membranes (Roche). Membranes were blocked (1 h, room temperature) in 5% fat-free milk (MARVEL) in TBS with 0.1% Tween-20 (TBST), incubated with the appropriate primary antibodies at 4 °C overnight followed by incubation with HRP-conjugated secondary antibodies (Dako) and imaged (BioSpectrum 810; UVP). Antibodies used were: SIRT5 (ATLAS ANTIBODIES, HPA022002; Cell Signaling, 8782), LC3-I/II (Cell Signaling, 4108; 2775), p62 (Cell Signaling, 5114), TOM20 (SANTA CRUZ, sc-17764; proteintech, 11802-1-AP), TIM23 (SANTA CRUZ, sc-514463), SDHA (SANTA CRUZ, sc-390381), VDAC1 (SANTA CRUZ, sc-390996), MFN1 (abcam, ab57602), MFN2 (abcam, ab56889), OPA1 (BD, 612606), DRP1 (SANTA CRUZ, sc-271583), DRP1-S616 (Cell Signaling, 3455), FIS1 (SANTA CRUZ, sc-376447), MiD51 (proteintech, 20164-1-AP), OMA1 (SANTA CRUZ, sc-515788), YME1L (proteintech, 11510-1-AP), Actin (SIGMA-ALDRICH, A2066) and Tubulin (abcam, ab52866). For details see Supplementary Table 2.

### Mitophagy detection

The Mitophagy Detection kit (DOJINDO) was used according to the manufacturer’s instructions. Transfected cells (control and *SIRT5* RNAi) were loaded with a pH-dependent, fluorescent probe (Mtphagy Dye), exposed to 6 h of normoxia in CM or OND. Lysosomes were then labelled using the Lyso Dye (DOJINDO). The fluorescence intensity of the Mtphagy Dye increases in an acidic environment, thus, the combination of Mtophagy and Lyso Dyes allows specific detection of lysosome-engulfed mitochondria (DOJINDO). Cells were imaged on a TCS SP8 confocal microscope (Leica) and analyzed with ImageJ. Integrated optical density (IOD) of Mtophagy Dye was assessed using Image-Pro Premier 3D (MEDIA CYBERNETICS).

### Citrate synthase assay

Cells were lysed in 0.1% Triton X-100 in PBS supplemented with Halt Protease and Phosphatase Inhibitor Cocktail (Thermo Fisher Scientific) and pepstatin A. Protein concentration was determined (DC Assay; BIO-RAD), with BSA as a standard. Citrate synthase (CS) activity was measured according to Srere et al.^76^ Briefly, acetyl Co-enzyme A (100 µM), oxalacetic acid (100 µM) and 5,5′-dithiobis(2-nitrobenzoic acid) were combined in Tris·HCl buffer (100 mM, pH 8.0) and the rate of 5-thio-2-nitrobenzoic acid formation was measured at 30 °C using a plate reader (412 nm; BioTek Synergy HT). CS activities were normalized to protein concentration/sample.

### Statistical analyses

Data were analyzed using GraphPad Prism 7.0 software. Parametric data are presented as mean ± SD and non-parametric data as median ± interquartile range (IQR) with *p* < 0.05 considered statistically significant. For n = 2 groups differences between groups were assessed using a Mann–Whitney U test. Where n > 2 groups differences between groups were measured using one-way or two-way ANOVA with Tukey’s post hoc test to correct for multiple comparisons.

## Supplementary Information

Supplementary Information.

## References

[1] Meersch M, Schmidt C, Zarbock A (2017). Perioperative acute kidney injury: An under-recognized problem. Anesth. Analg..

[2] Emma F, Montini G, Parikh SM, Salviati L (2016). Mitochondrial dysfunction in inherited renal disease and acute kidney injury. Nat. Rev. Nephrol..

[3] Twig G (2008). Fission and selective fusion govern mitochondrial segregation and elimination by autophagy. EMBO J..

[4] Wai T, Langer T (2016). Mitochondrial dynamics and metabolic regulation. Trends Endocrinol. Metab..

[5] Rambold AS, Kostelecky B, Elia N, Lippincott-Schwartz J (2011). Tubular network formation protects mitochondria from autophagosomal degradation during nutrient starvation. Proc. Natl. Acad. Sci. U. S. A..

[6] Tilokani L, Nagashima S, Paupe V, Prudent J (2018). Mitochondrial dynamics: Overview of molecular mechanisms. Essays Biochem..

[7] Loson OC, Song Z, Chen H, Chan DC (2013). Fis1, Mff, MiD49, and MiD51 mediate Drp1 recruitment in mitochondrial fission. Mol. Biol. Cell.

[8] Szeto HH (2017). Mitochondria protection after acute ischemia prevents prolonged upregulation of IL-1beta and IL-18 and arrests CKD. J. Am. Soc. Nephrol..

[9] Brooks C, Wei Q, Cho SG, Dong Z (2009). Regulation of mitochondrial dynamics in acute kidney injury in cell culture and rodent models. J. Clin. Investig..

[10] Plotnikov EY (2008). Interrelations of mitochondrial fragmentation and cell death under ischemia/reoxygenation and UV-irradiation: protective effects of SkQ1, lithium ions and insulin. FEBS Lett..

[11] Parekh DJ (2013). Tolerance of the human kidney to isolated controlled ischemia. J. Am. Soc. Nephrol..

[12] Plotnikov EY (2007). The role of mitochondria in oxidative and nitrosative stress during ischemia/reperfusion in the rat kidney. Kidney Int..

[13] Perry HM (2018). Dynamin-related protein 1 deficiency promotes recovery from AKI. J. Am. Soc. Nephrol..

[14] Hall AM, Unwin RJ, Parker N, Duchen MR (2009). Multiphoton imaging reveals differences in mitochondrial function between nephron segments. J. Am. Soc. Nephrol..

[15] Lan R (2016). Mitochondrial pathology and glycolytic shift during proximal tubule atrophy after ischemic AKI. J. Am. Soc. Nephrol..

[16] Dickman KG, Mandel LJ (1990). Differential effects of respiratory inhibitors on glycolysis in proximal tubules. Am. J. Physiol..

[17] Hershberger KA, Martin AS, Hirschey MD (2017). Role of NAD(+) and mitochondrial sirtuins in cardiac and renal diseases. Nat. Rev. Nephrol..

[18] Fan H (2013). The histone deacetylase, SIRT1, contributes to the resistance of young mice to ischemia/reperfusion-induced acute kidney injury. Kidney Int..

[19] Morigi M (2015). Sirtuin 3-dependent mitochondrial dynamic improvements protect against acute kidney injury. J. Clin. Investig..

[20] Zhao WY, Zhang L, Sui MX, Zhu YH, Zeng L (2016). Protective effects of sirtuin 3 in a murine model of sepsis-induced acute kidney injury. Sci. Rep..

[21] Peng C (2011). The first identification of lysine malonylation substrates and its regulatory enzyme. Mol. Cell. Proteomics.

[22] Du J (2011). Sirt5 is a NAD-dependent protein lysine demalonylase and desuccinylase. Science.

[23] Tan M (2014). Lysine glutarylation is a protein posttranslational modification regulated by SIRT5. Cell Metab..

[24] Boylston JA (2015). Characterization of the cardiac succinylome and its role in ischemia–reperfusion injury. J. Mol. Cell. Cardiol..

[25] Rardin MJ (2013). SIRT5 regulates the mitochondrial lysine succinylome and metabolic networks. Cell Metab..

[26] Nishida Y (2015). SIRT5 regulates both cytosolic and mitochondrial protein malonylation with glycolysis as a major target. Mol. Cell.

[27] Sadhukhan S (2016). Metabolomics-assisted proteomics identifies succinylation and SIRT5 as important regulators of cardiac function. Proc. Natl. Acad. Sci. U. S. A..

[28] Guedouari H, Daigle T, Scorrano L, Hebert-Chatelain E (2017). Sirtuin 5 protects mitochondria from fragmentation and degradation during starvation. Biochim. Biophys. Acta Mol. Cell Res..

[29] Zhu WZ, Wu XF, Zhang Y, Zhou ZN (2012). Proteomic analysis of mitochondrial proteins in cardiomyocytes from rats subjected to intermittent hypoxia. Eur. J. Appl. Physiol..

[30] Morris-Blanco KC (2016). Protein kinase C epsilon promotes cerebral ischemic tolerance via modulation of mitochondrial Sirt5. Sci. Rep..

[31] Xie LP, Zheng XY, Qin J, Tong YY (2004). Amino acids protects against renal ischemia–reperfusion injury and attenuates renal endothelin-1 disorder in rats. Chin. J. Traumatol..

[32] Weinberg JM (1990). The effect of amino acids on ischemic and toxic injury to the kidney. Semin. Nephrol..

[33] Jiang M, Liu K, Luo J, Dong Z (2010). Autophagy is a renoprotective mechanism during in vitro hypoxia and in vivo ischemia–reperfusion injury. Am. J. Pathol..

[34] Nicholls DG (2004). Mitochondrial membrane potential and aging. Aging Cell.

[35] Cereghetti GM (2008). Dephosphorylation by calcineurin regulates translocation of Drp1 to mitochondria. Proc. Natl. Acad. Sci. U. S. A..

[36] Rainbolt TK, Lebeau J, Puchades C, Wiseman RL (2016). Reciprocal degradation of YME1L and OMA1 adapts mitochondrial proteolytic activity during stress. Cell Rep..

[37] Anand R (2014). The i-AAA protease YME1L and OMA1 cleave OPA1 to balance mitochondrial fusion and fission. J. Cell Biol..

[38] Ishihara N, Fujita Y, Oka T, Mihara K (2006). Regulation of mitochondrial morphology through proteolytic cleavage of OPA1. EMBO J..

[39] Lee H, Smith SB, Yoon Y (2017). The short variant of the mitochondrial dynamin OPA1 maintains mitochondrial energetics and cristae structure. J. Biol. Chem..

[40] MacVicar T, Langer T (2016). OPA1 processing in cell death and disease—The long and short of it. J. Cell Sci..

[41] Baburamani AA (2015). Mitochondrial optic atrophy (OPA) 1 processing is altered in response to neonatal hypoxic-ischemic brain injury. Int. J. Mol. Sci..

[42] Trump BF (1975). The application of electron microscopy and cellular biochemistry to the autopsy. Observations on cellular changes in human shock. Hum. Pathol..

[43] Mergner WJ, Chang SH, Trump BF (1976). Studies on the pathogenesis of ischemic cell injury. V. Morphologic changes of the pars convoluta (P1 and P2) of the proximal tubule of rat kidney made ischemic in vitro. Virchows Arch. B Cell Pathol..

[44] Birk AV (2013). The mitochondrial-targeted compound SS-31 re-energizes ischemic mitochondria by interacting with cardiolipin. J. Am. Soc. Nephrol..

[45] Zoratti M, Szabo I (1995). The mitochondrial permeability transition. Biochim. Biophys. Acta.

[46] Halestrap AP (1989). The regulation of the matrix volume of mammalian mitochondria in vivo and in vitro and its role in the control of mitochondrial metabolism. Biochim. Biophys. Acta.

[47] Gomes LC, Scorrano L (1833). Mitochondrial morphology in mitophagy and macroautophagy. Biochim. Biophys. Acta.

[48] Larsen S (2012). Biomarkers of mitochondrial content in skeletal muscle of healthy young human subjects. J. Physiol..

[49] Padanilam BJ (2003). Cell death induced by acute renal injury: A perspective on the contributions of apoptosis and necrosis. Am. J. Physiol. Ren. Physiol..

[50] Szeto HH (2011). Mitochondria-targeted peptide accelerates ATP recovery and reduces ischemic kidney injury. J. Am. Soc. Nephrol..

[51] Decleves AE, Sharma K, Satriano J (2014). Beneficial effects of AMP-activated protein kinase agonists in kidney ischemia–reperfusion: Autophagy and cellular stress markers. Nephron Exp. Nephrol..

[52] Chang YK (2016). Dapagliflozin, SGLT2 inhibitor, attenuates renal ischemia–reperfusion injury. PLoS One.

[53] Seo-Mayer PW (2011). Preactivation of AMPK by metformin may ameliorate the epithelial cell damage caused by renal ischemia. Am. J. Physiol. Ren. Physiol..

[54] Chouchani ET (2014). Ischaemic accumulation of succinate controls reperfusion injury through mitochondrial ROS. Nature.

[55] Atwal KS (2004). Small molecule mitochondrial F1F0 ATPase hydrolase inhibitors as cardioprotective agents. Identification of 4-(*N*-arylimidazole)-substituted benzopyran derivatives as selective hydrolase inhibitors. J. Med. Chem..

[56] Yamamoto S (2020). Spatiotemporal ATP dynamics during AKI predict renal prognosis. J. Am. Soc. Nephrol..

[57] Li W, Yang Y, Li Y, Zhao Y, Jiang H (2019). Sirt5 attenuates cisplatin-induced acute kidney injury through regulation of Nrf2/HO-1 and Bcl-2. Biomed. Res. Int..

[58] Ishihara N, Eura Y, Mihara K (2004). Mitofusin 1 and 2 play distinct roles in mitochondrial fusion reactions via GTPase activity. J. Cell Sci..

[59] Lim KH (2002). The effects of ischaemic preconditioning, diazoxide and 5-hydroxydecanoate on rat heart mitochondrial volume and respiration. J. Physiol..

[60] Yu J (2013). Metabolic characterization of a Sirt5 deficient mouse model. Sci. Rep..

[61] Schmiedl A, Schnabel PA, Richter J, Bretschneider HJ (1993). Close correlations between mitochondrial swelling and ATP-content in the ischemic canine myocardium. A combined morphometric and biochemical study. Pathol. Res. Pract..

[62] Chiba T (2019). Sirtuin 5 regulates proximal tubule fatty acid oxidation to protect against AKI. J. Am. Soc. Nephrol..

[63] Zhang W (2018). Rotenone ameliorates chronic renal injury caused by acute ischemia/reperfusion. Oncotarget.

[64] Tanaka R (2013). Oligomycin, an F1Fo-ATPase inhibitor, protects against ischemic acute kidney injury in male but not in female rats. J. Pharmacol. Sci..

[65] Kalakeche R (2011). Endotoxin uptake by S1 proximal tubular segment causes oxidative stress in the downstream S2 segment. J. Am. Soc. Nephrol..

[66] Usuda N, Yokota S, Hashimoto T, Nagata T (1986). Immunocytochemical localization of D-amino acid oxidase in the central clear matrix of rat kidney peroxisomes. J. Histochem. Cytochem..

[67] El-Achkar TM, Dagher PC (2015). Tubular cross talk in acute kidney injury: A story of sense and sensibility. Am. J. Physiol. Ren. Physiol..

[68] Zhuo JL, Li XC (2013). Proximal nephron. Compr. Physiol..

[69] Zhang Y (2017). Lysine desuccinylase SIRT5 binds to cardiolipin and regulates the electron transport chain. J. Biol. Chem..

[70] Fisher-Wellman KH (2019). Respiratory phenomics across multiple models of protein hyperacylation in cardiac mitochondria reveals a marginal impact on bioenergetics. Cell Rep..

[71] Hesketh EE (2014). Renal ischaemia reperfusion injury: A mouse model of injury and regeneration. J. Vis. Exp..

[72] Racusen LC (1997). Cell lines with extended in vitro growth potential from human renal proximal tubule: Characterization, response to inducers, and comparison with established cell lines. J. Lab. Clin. Med..

[73] Kang HM (2015). Defective fatty acid oxidation in renal tubular epithelial cells has a key role in kidney fibrosis development. Nat. Med..

[74] Zhao J (2018). Genomic integration of ERRgamma-HNF1beta regulates renal bioenergetics and prevents chronic kidney disease. Proc. Natl. Acad. Sci. U. S. A..

[75] Valente AJ, Maddalena LA, Robb EL, Moradi F, Stuart JA (2017). A simple ImageJ macro tool for analyzing mitochondrial network morphology in mammalian cell culture. Acta Histochem..

[76] Srere, P. A. In *Citric Acid Cycle**Vol. 13 Methods in Enzymology* 3–11 (Academic Press, 1969).

