# Supplementary material for: Sirtuin 5 depletion impairs mitochondrial function in human proximal tubular epithelial cells
Source: Sci Rep. 2021 Jul 30;11:15510. doi: 10.1038/s41598-021-94185-6 (PMC8324880; doi:10.1038/s41598-021-94185-6)
Supplement: Supplementary file 1 — Supplementary Information. [file 41598_2021_94185_MOESM1_ESM.pdf]

Supplementary Material for

Sirtuin 5 depletion impairs mitochondrial function in human proximal tubular epithelial cells

Timo N. Haschler[1*], Harry Horsley[1], Monika Balys[4], Glenn Anderson[4], Jan-Willem Taanman[3], Robert J. Unwin[1,2], Jill T. Norman[1]

[1] Department of Renal Medicine, UCL Medical School, Royal Free Campus, London, UK.

[2] Early Clinical Development, Research and Early Development, Cardiovascular, Renal and Metabolism (CVRM), BioPharmaceuticals R&D, AstraZeneca, Cambridge, UK.

[3] Department of Clinical & Movement Neurosciences, UCL Queen Square Institute of Neurology, Royal Free Campus, London, UK.

[4] Department of Histopathology, Great Ormond Street Hospital, London, UK.

*Corresponding author: Timo N. Haschler, Department of Renal Medicine, 2nd floor UCL Medical School, Royal Free Campus, Rowland Hill Street, London NW3 2PF, UK
Email: timo.haschler.16@ucl.ac.uk; **Tel:** +44 20 7794 0500

This PDF file includes:

Supplementary methods
Figures S1 – S9
Tables S1 - S3

Supplementary Material

Methods

***In vitro* ischemia.** HKC-8 cells (hPTECs) were seeded in 6-well plates (Corning) at a density of 6x10^5 cells/well. After 24 hours, cells were 90% confluent and were used for experiments. To mimic ischemia *in vitro* a combined oxygen and nutrient-deprivation (OND) model was developed, which implements both aspects of ischemia *in vivo* i.e. hypoxia and nutrient starvation. To differentiate between the impact of (i) oxygen deprivation (OD; hypoxia) and (ii) nutrient deprivation (ND; starvation), hPTECs were cultured in complete medium (CM; DMEM/F12 (gibco) supplemented with 5% Fetal Bovine Serum (FBS; Seralab)) or in starvation medium (Hank's balanced salt solution (HBSS); gibco) supplemented with 10mM HEPES (pH 7.4; gibco) under either normoxia (21%O_2, 5%CO_2, 74%N_2) or hypoxia (1%O_2, 5%CO_2, 94%N_2; BOC) and harvested after 6 or 24 hours.

SIRT5 siRNA knockdown. SIRT5 expression was reduced by RNA interference (RNAi) using the SMARTpool:ON-TARGETplus system (Dharmacon). hPTECs were transfected with either the small interfering RNA (siRNA) pool against *SIRT5* (L-013448-01-0005, 50nM) or the control siRNA pool (ON-TARGETplus Non-targeting Pool, D-001810-10-05, 50nM) using Dharmafect-1 (Dharmacon) according to the manufacturer's protocol. To reduce the toxicity of the transfection reagent, the medium was changed 24 hours post-transfection. Knockdown efficiency was determined by Western blotting (WB) and 72 hours post-transfection was chosen as the optimal time-point with >85% knockdown.

For WB, fluorescence-activated cell sorting (FACS) and mitochondrial structure analyses (confocal and transmission electron microscopy (TEM)), hPTECs were seeded in T25

flasks (Corning) at a density of 1.5x10^6 cells/flask and transfected the following day. Twenty-four hours post-transfection, transfection medium was changed to CM to reduce the cytotoxicity of the transfection reagent. Forty-eight hours post-transfection, cells were re-plated in 6-well plates (WB and FACS analyses; 6x10^5 cells/well), 8-well chamber slides (Nunc Lab-Tek) (confocal microscopy; 5x10^4 cells/well), 10cm petri dishes (Corning) (TEM; 4.5x10^5 cells/dish), 8-well Seahorse XFp plates (Agilent) (respirometry; 2.5x10^4 cells/well) or 96-well clear bottom plates (Corning) (2.5x10^4 cells/well) for viability measurements (WST-1; Abcam, ab65473). For ATP quantification, hPTECs were seeded into 96-well white clear bottom plates (Corning), medium was changed 24 hours post-transfection and the assay was carried out 72 hours post-transfection without re-plating.

Ultrastructural analysis of mitochondria. hPTECs (control RNAi- and *SIRT5* RNAi-treated) were seeded in 10cm petri dishes (Corning) at a density of 4.5x10^6 cells/plate. The following day, cells underwent either OND or were cultured in CM under normoxic conditions (control; protocol described in "*In vitro* ischemia") for 6 hours. Cells were washed with PBS (gibco), detached from the culture plates using 3ml 0.05% Trypsin-EDTA (gibco) and suspended in 7ml CM for trypsin inactivation. Cells were centrifuged for 5 minutes at 400xg at room temperature (RT), supernatants removed and the pellets washed with PBS. After centrifugation for 5 minutes at 400xg, supernatants were aspirated and cells fixed in 5ml 2.5% glutaraldehyde buffered with 100mM sodium cacodylate (pH 7.2; agar scientific) for 24 hours at RT and stored for up to a week at 4ºC. Sample processing and imaging were performed by the Histopathology Department (Great Ormond Street Hospital, London). Briefly, after secondary fixation in 1% osmium tetroxide (agar scientific) for 2 hours, samples were dehydrated in graded ethanols, transferred to propylene oxide (agar scientific) then infiltrated and embedded in Agar 100 epoxy resin (agar scientific). Polymerisation was carried out at 60°C for 48 hours. Ultrathin sections

(90nm) were cut using a Diatome diamond knife on a Leica EM UC7 ultramicrotome. Sections were collected on copper grids and stained with alcoholic uranyl acetate (agar scientific) and lead citrate (agar scientific). Sections were examined with a JEOL 1400 TEM and digital images recorded using an AMT XR80 digital camera.

Assessment of mitochondrial function using respirometry. Oxygen consumption rate (OCR; a measure of mitochondrial function) and extracellular acidification rate (ECAR; a measure of glycolysis) were assessed using a Seahorse XFp Analyzer (Agilent). hPTECs (control RNAi- and *SIRT5* RNAi-treated) were seeded in Seahorse XFp 8-well plates in CM and grown overnight. Cells were exposed to 6 hours OND. After OND, HBSS was replaced by HEPES-buffered DMEM (Agilent) and plates were incubated in a humidified chamber without CO_2 for 1 hour at 37°C to stabilize pH and temperature. Sensor cartridges (Agilent) required for mitochondrial toxin injection were hydrated the day before the assay and kept in a humidified incubator without CO_2 at 37°C. After assessment of basal respiration, oligomycin (2μM; a mitochondrial ATP-synthase inhibitor; gives insight into proton leak), Carbonyl cyanide-4-phenylhydrazone (FCCP) (0.75μM; a mitochondrial uncoupler; reveals maximal cellular respiration) and rotenone (1μM) with antimycin A (1μM; electron transport chain complex 1 and 3 inhibitors; inhibition of mitochondrial respiration to analyze non-mitochondrial respiration) were sequentially injected. Measurement of OCR and ECAR was carried out every 3 minutes for 2 hours. OCR and ECAR were normalized to total cell number/well (CyQUANT; Life Technologies). Data were analysed using the Wave software (Agilent).

Crude mitochondrial isolation. Kidneys from WT and *Sirt5*$^{-/-}$ mice were decapsulated, chopped into small pieces (1mm^3), transferred to pre-cooled glass

tubes and isotonic homogenization buffer (0.32M sucrose, 1mM EDTA, 10mM Tris-HCl; pH 7.4) was added (total volume of 2ml). Tissues were homogenized using a Potter Elvehejm tissue homogenizer (Eurostar). The homogenate was transferred to a 50ml centrifuge tube (Corning) and centrifuged at 1500xg for 10min at 4ºC. The mitochondria-containing supernatant was transferred to a new 50ml tube and centrifuged at 11,500xg for 10min at 4ºC. The supernatant was removed and the pellets re-suspended in 500µl homogenization buffer. Crude mitochondrial extracts were flash-frozen in liquid N_2 and stored at -80ºC until analyzed.

Complex II activity assay. Succinate ubiquinone oxidoreductase (complex II) activity was measured in crude mitochondrial extracts from WT and *Sirt5*$^{-/-}$ kidneys. A modified version based on the method of Hatefi and Stiggall [1] was used. This assay measures the succinate-ubiquinone reductase activity indirectly through the CoQ2 (ubiquinone-2)-dependent reduction of 2,6-dichloroindophenol (DCIP), a blue dye which shows maximal absorption at 600nm. The time-dependent decrease in DCIP absorption allows the determination of complex II activity. Before complex II activity could be assessed, mitochondrial extracts were subjected to three freeze-thaw cycles to release mitochondrial enzymes: Samples were flash-frozen in liquid nitrogen and subsequently thawed in a water-bath at 30°C for 2 minutes.

To determine complex II activity, absorption was determined in a test cuvette and compared to the absorption of a reference cuvette. An assay solution was prepared: potassium phosphate buffer (50mM; K_2HPO_4; KH_2PO_4), EDTA (100µM), DCIP (74µM), sodium-succinate (20mM), sodium-cyanide (1mM; complex IV inhibitor which prevents

electron flow to O_2), rotenone (10μM; complex I inhibitor which prevents electron flow along complex I). The reference cuvette contained the assay solution and in addition, the complex II inhibitor 2-thenoyltrifluoroacetone (TTFA; 1mM) and the sample (shows succinate-dependent, CoQ2-independent reduction of DCIP). The test cuvette also contained the assay buffer. Sequential addition of sample, CoQ2 and TTFA allowed determination of complex II activity. The detailed process was carried out as follows: (1) Assay solution was added to the cuvette. (2) At t=50s, sample was added to the assay solution, mixed carefully and absorption was measured at t=120s and t=180s to determine the succinate-dependent, but CoQ2-independent reduction of DCIP (background). (3) At t=200s, CoQ2 (50μM; Sigma®) was added to the assay solution, mixed thoroughly and absorption measured at t=240s and t=360s (CoQ2-dependent and -independent reduction of DCIP). (4) Finally, TTFA was added which inhibits complex II activity. Based on the molar extinction coefficient of DCIP (21,000 $M^{-1}\cdot cm^{-1}$) the rate of the complex II activity can be determined:

$$\text{Rate } (\mu\text{mol/min/mg}) = \frac{\frac{\text{Absorption}}{\text{time}}}{21000} \text{ x } \frac{1{,}000{,}000}{\text{Protein conc.}}$$

A Hitachi U-3310 UV-Vis spectrophotometer was used to measure absorption of DCIP. All measurements were carried out in quadruplicate at 30°C. Complex II enzyme activities were normalised to the total protein content of the sample as determined by the DC protein assay (Bio-Rad).

WST-1 assay. hPTECs were incubated with the WST-1 reagent (Abcam, ab65473) for 2h and absorbance measured at 450nm (formazan dye) and 650nm (background) using a Mithras LB 940 Multimode plate reader (Berthold Technologies). Absorbance = $A_{450} - A_{650}$

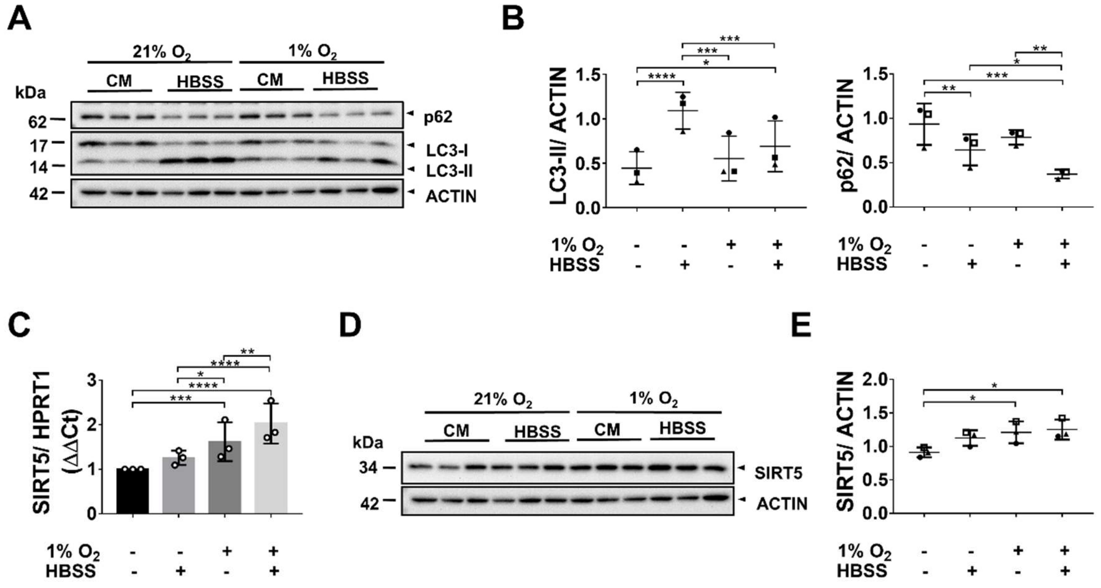

Fig. S1. OND induces autophagy in hPTECs. hPTECs were exposed to hypoxia (1%O_2) or normoxia (21%O_2) with or without nutrient depletion (HBSS) for 24 hours. **(A)** WB showing protein levels of autophagy markers p62 and LC3. (**B**) Scatter plots showing densitometry of LC3-II and p62 protein expression. **(C)** Bar graph showing mRNA expression of *SIRT5*. **(D)** WBs showing protein levels of SIRT5. **(E)** Scatter plot of densitometry of SIRT5 protein expression. Protein levels of p62 and LC3 were normalized to actin. ImageJ was used for densitometric analysis. Whole WBs shown in supplementary file (Fig. S8h). Data are from three independent experiments with three technical replicates/group. Data are mean ± SD. To determine statistical significance of the protein data, a two-way ANOVA was carried out followed by Tukey's *post hoc* test to normalize for multiple comparisons. *p<0.05, **p<0.01, ***p<0.001 and ****p<0.0001.

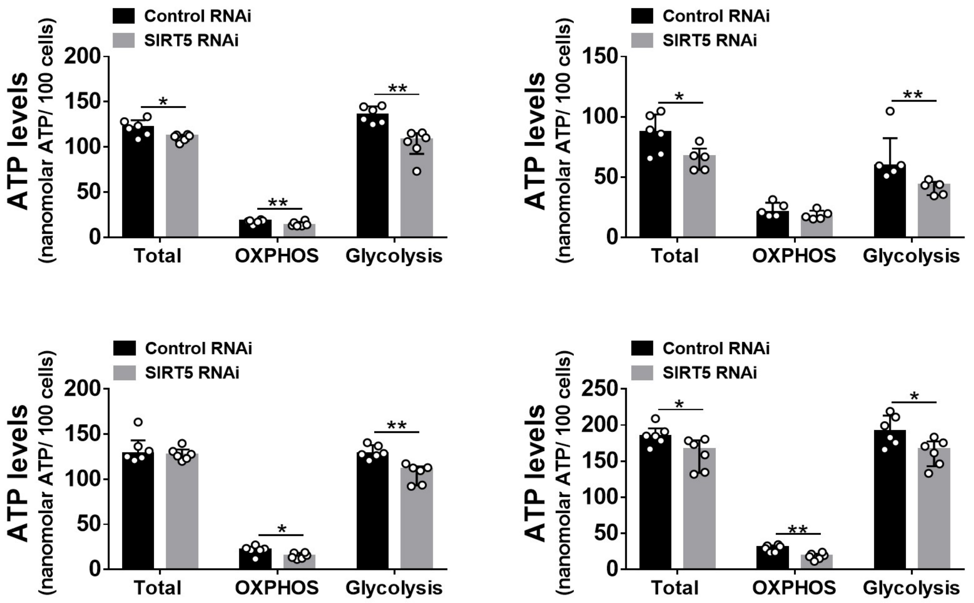

Fig. S2. SIRT5 depletion impairs cellular bioenergetics. *SIRT5* knockdown by RNAi was carried out in hPTECs with non-targeting siRNA as control. All experiments were performed 72 hours after siRNA transfection. Bar graphs show ATP levels. hPTECs (control and *SIRT5* RNAi) were analysed without inhibitor treatment to determine total ATP levels (glycolytic + mitochondrial ATP; 2-deoxyglucoe and oligomycin A), after treatment with 2-deoxyglucose to inhibit glycolysis (mitochondrial ATP; OXPHOS) or after treatment with oligomycin A to inhibit mitochondrial ATP synthase (glycolytic ATP; Glycolysis). ATP levels were normalized to cell number as measured by DNA quantification. Data shown are four independent experiments with n=5-6 technical replicates/group. Data are median ± IQR. To determine statistical significance a Mann-Whitney U test was applied. *p<0.05 and **p<0.01.

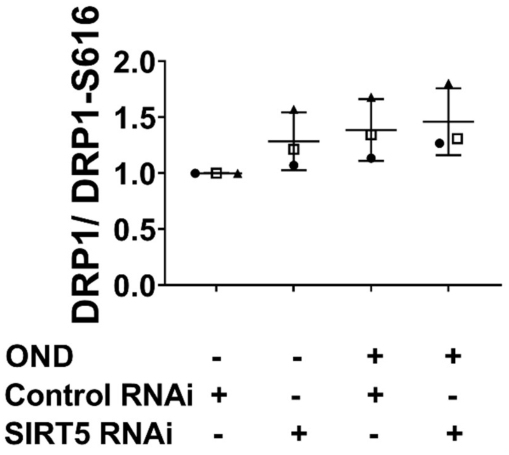

Fig. S3 SIRT5-depletion does not affect the DRP1-S616/ DRP1 ratio. *SIRT5* knockdown by RNAi was carried out in hPTECs with non-targeting siRNA as control. Seventy-two hours post-transfection, cells were exposed to 6 hours OND (1%O_2+HBSS) or normoxia and complete medium (21%O_2+CM). Scatter plots showing densitometry of DRP1-S616 protein expression normalized to total DRP1 protein expression. ImageJ was used for densitometric analysis. Data are from three independent experiments with three technical replicates/group. Data are mean ± SD. To determine statistical significance a two-way ANOVA was carried out followed by Tukey's *post hoc* test to normalize for multiple comparisons.

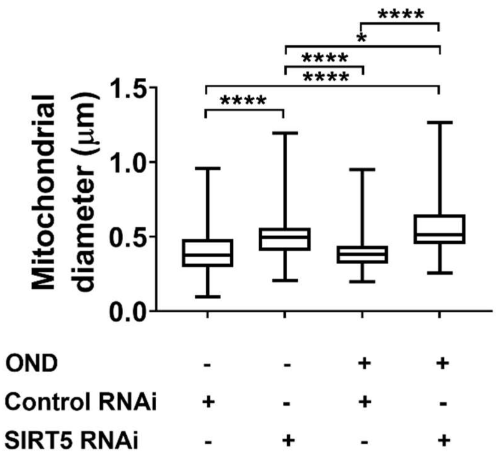

Fig. S4. Quantitative assessment of *SIRT5* depletion-induced mitochondrial swelling. hPTECs were transfected with *SIRT5* siRNA or non-targeting siRNA control. Seventy-two hours post-transfection, cells were exposed to 6 hours OND (1%O_2+HBSS) or normoxia and complete medium (21%O_2+CM). Box and whisker plots display mitochondrial diameter (in µm) as median interquartile range and minimum and maximum values. Between 162-273 mitochondria from >10 cells/treatment group were analysed using ImageJ. To determine statistical significance a two-way ANOVA was carried out followed by Tukey's *post hoc* test to normalize for multiple comparisons. *p<0.05 and ****p<0.0001.

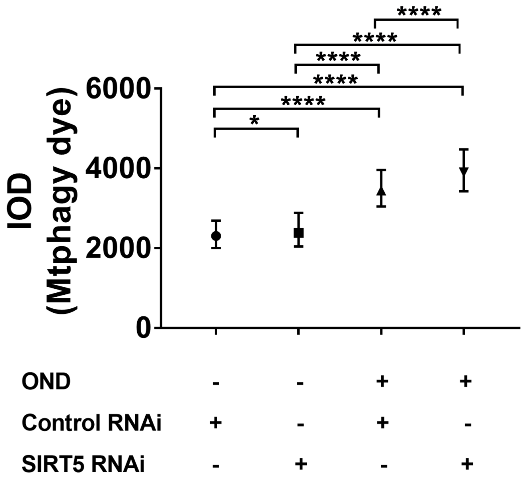

Fig. S5. Quantitative assessment of integrated optical density of Mtphagy dye. hPTECs were transfected using siRNA against *SIRT5* or non-targeting siRNA control. Seventy-two hours post-transfection cells were exposed to 6 hours of OND (1%O2+HBSS) or normoxia and complete medium (21%O2+CM). Integrated optical density (IOD) of Mtophagy dye was assessed using Image-Pro Premier 10 (Media Cybernetics). Data are from two independent experiments and a total of >40 cells were analysed/condition. Data are median ± IQR. To determine statistical significance a Kruskal-Wallis test was carried out followed by Dunn's *post hoc* test to normalize for multiple comparisons. *p<0.05 and ****p<0.0001.

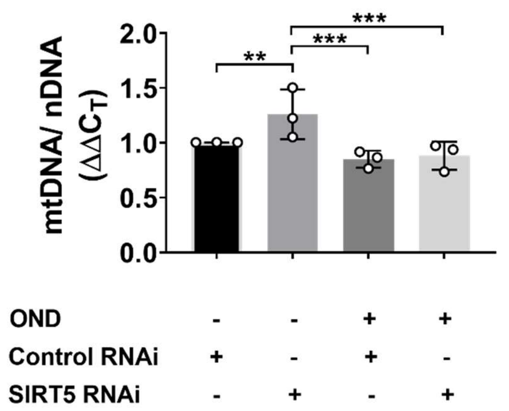

Fig. S6. Effect of *SIRT5* knockdown on mtDNA levels. hPTECs were transfected using *SIRT5* siRNA or non-targeting siRNA control. Seventy-two hours post-transfection cells were exposed to 6 hours OND (1%O_2+HBSS) or normoxia and complete medium (21%O_2+CM) as control. Bar graph showing mitochondrial DNA (mtDNA) copy number relative to nuclear DNA (nDNA; β2-microglobulin gene). $\Delta\Delta C_T$ was used to determine levels of mtDNA. Data are from three to four independent *SIRT5* knockdown experiments with n=3 technical replicates/group. Data are mean ± SD. To determine statistical significance a two-way ANOVA was carried out followed by Tukey's post hoc test to normalize for multiple comparisons. *p<0.05, **p<0.01, ***p<0.001 and ****p<0.0001.

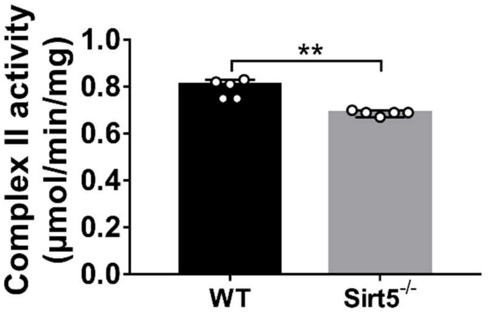

Fig. S7. Loss of SIRT5 impairs complex II activity. Complex II activity was measured in crude mitochondrial extracts from male WT and *Sirt5*$^{-/-}$ kidneys (8-10 weeks, n=5/group). Bar graph showing complex II activity. Data are median ± IQR. To determine statistical significance a Mann Whitney U test was carried out. **p<0.01.

Fig. 8: Original blots

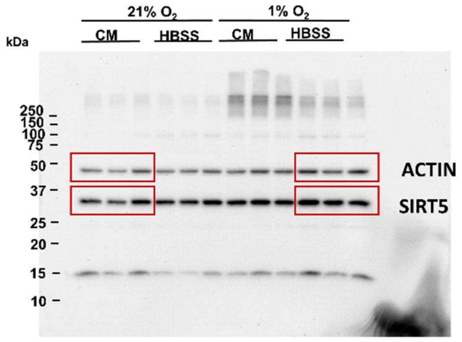

Fig. S8a. Effect of starvation, hypoxia and OND on SIRT5 protein expression.

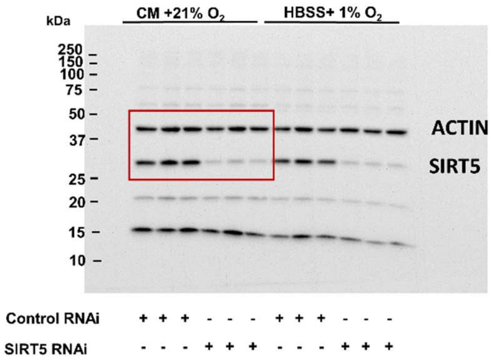

Fig. S8b. Knockdown of SIRT5 by siRNA.

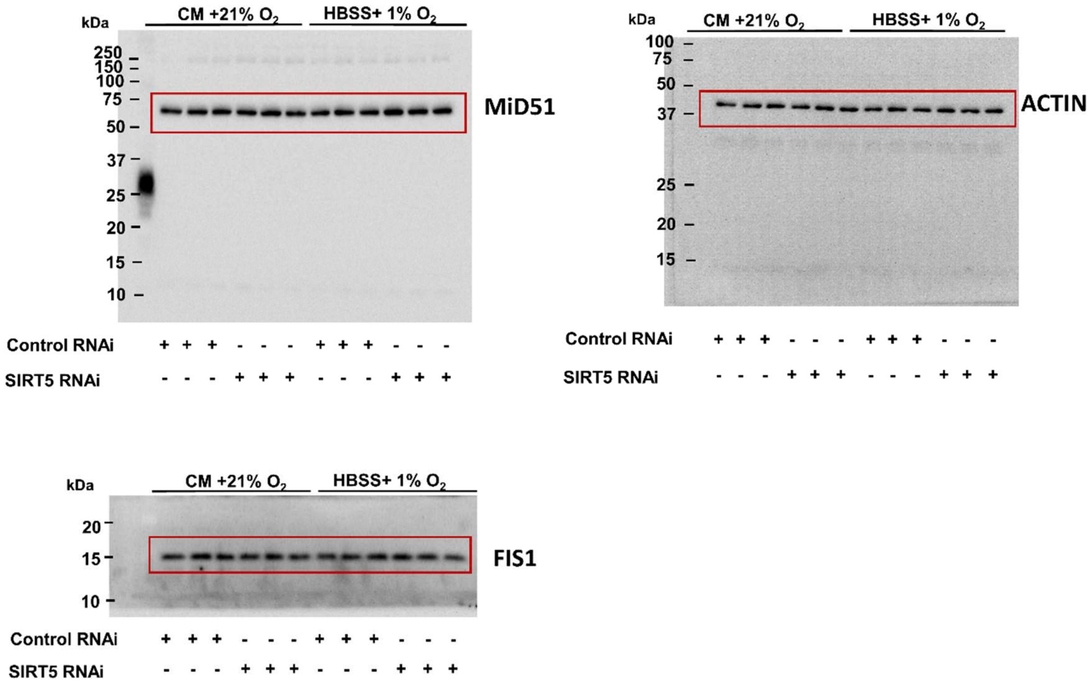

Fig. S8c. Effect *SIRT5* RNAi and OND on MiD51 and FIS1 protein expression.

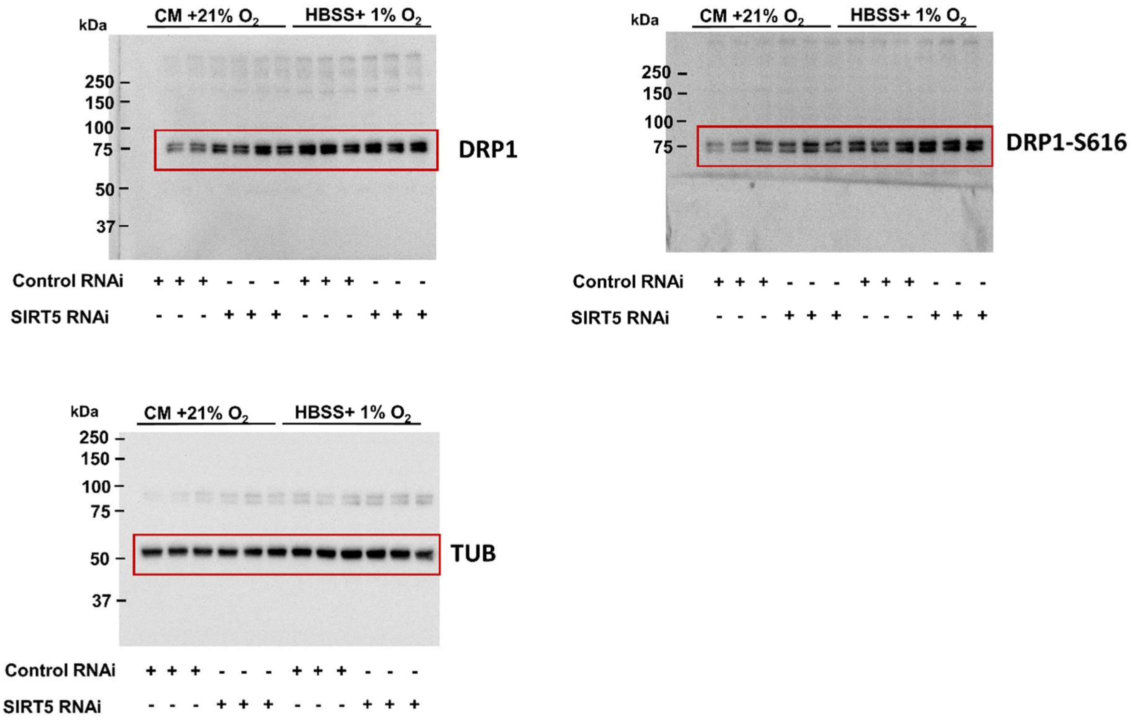

Fig. S8d. Effect *SIRT5* RNAi and OND on DRP1 and DPR1-S616 levels.

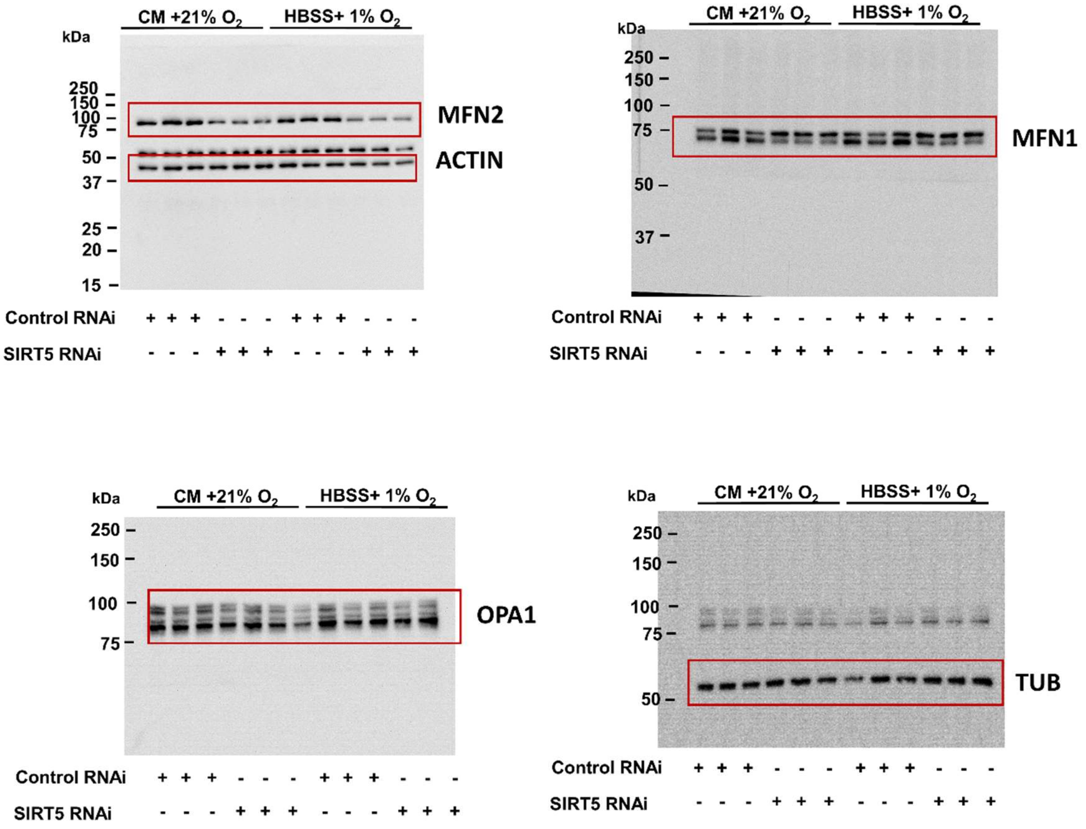

Fig. S8e. Effect *SIRT5* RNAi and OND on MFN1, MFN2 and OPA1 protein expression.

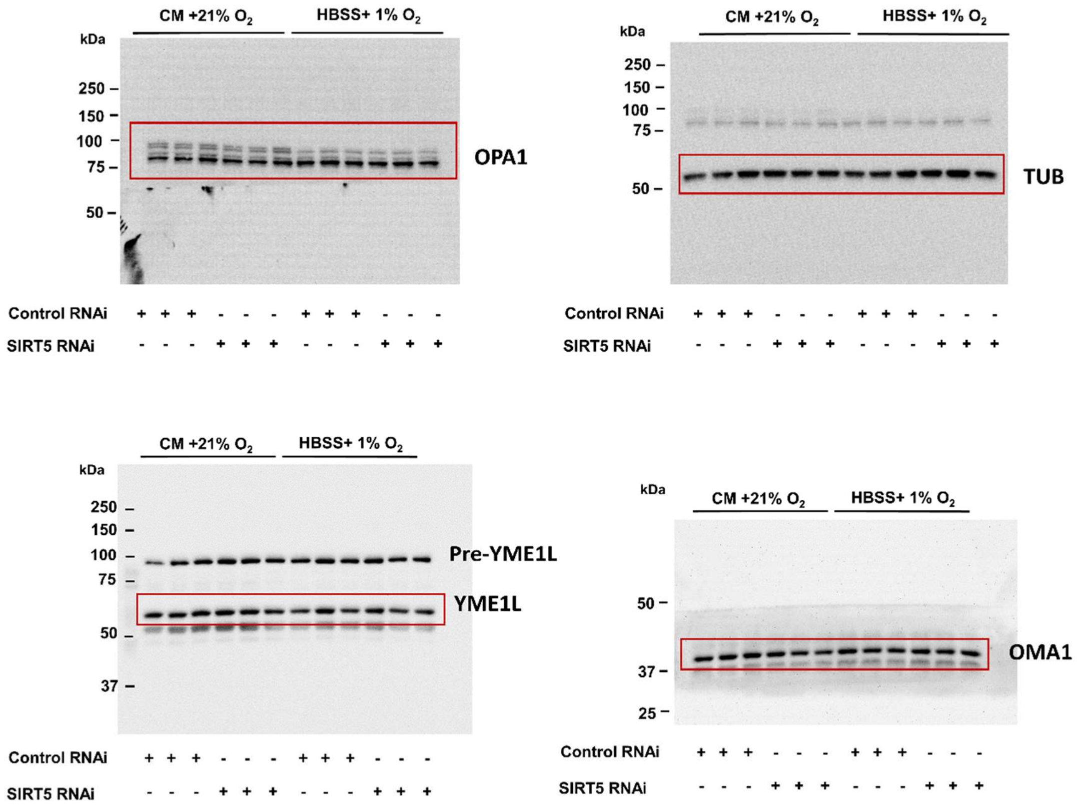

Fig. S8f. Effect *SIRT5* RNAi and OND on OPA1, YME1L and OMA1 protein expression.

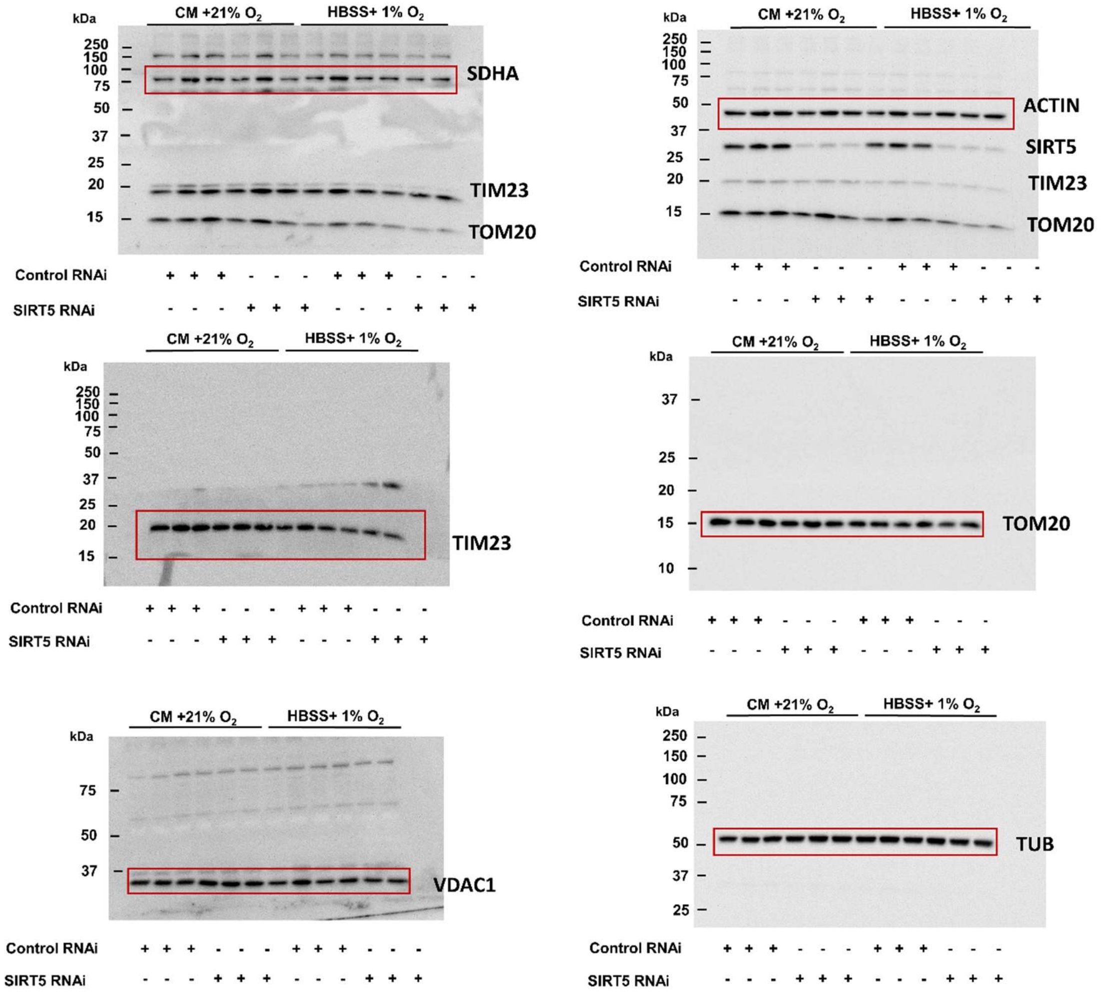

Fig. S8g. Effect *SIRT5* RNAi and OND on SDHA, TIM23, TOM20 and VDAC1 protein expression.

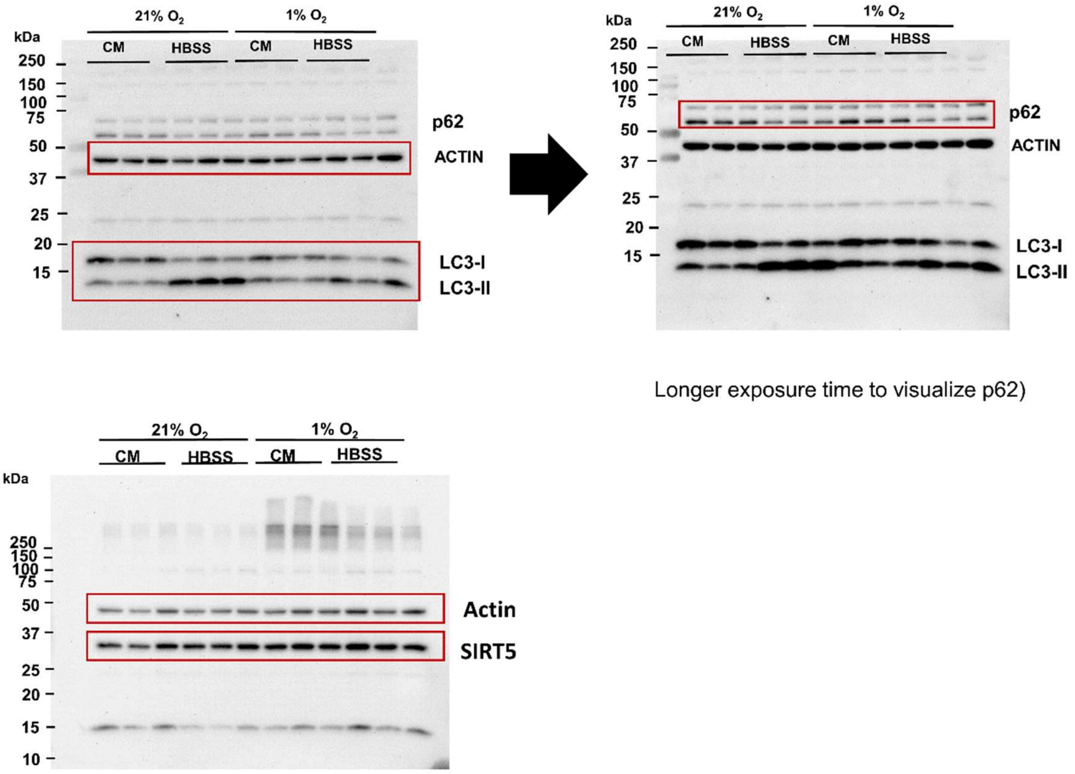

Fig. S8h. Effect hypoxia, starvation and OND on p62, LC3-I/II and SIRT5 protein levels.

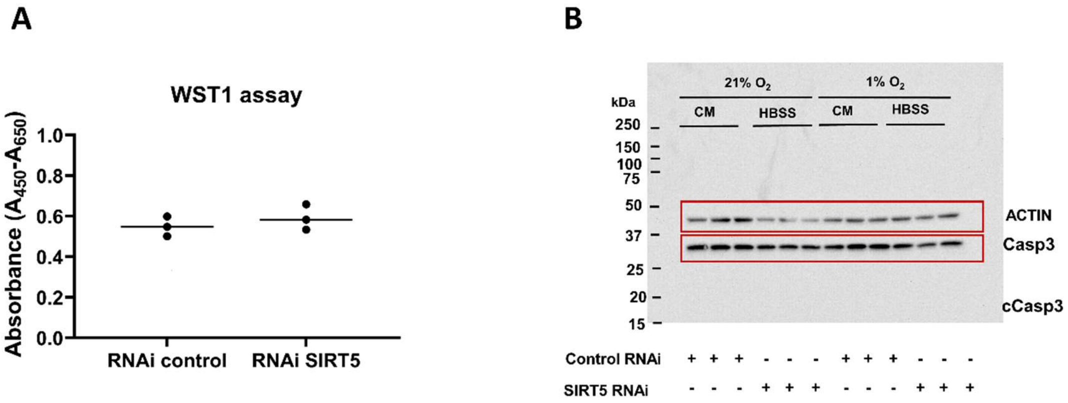

Fig. S9. *SIRT5* knockdown does not affect hPTEC viability. hPTECs were transfected using *SIRT5* siRNA or non-targeting siRNA control. (A) Seventy-two hours post-transfection a WST-1 assay performed. There was no difference in viability between SIRT5 RNAi and control RNAi-treated hPTECs. Data are median ± IQR. To determine statistical significance a Mann-Whitney U test was applied. (B) Seventy-two hours post-transfection cells were exposed to 6 hours OND (1%O_2+HBSS) or normoxia and complete medium (21%O_2+CM) as control. WB showing protein levels of caspase 3 (CASP3) and ACTIN.

Table S1: qPCR primers for mRNA and mtDNA expression analyses.

Gene	Direction (5'→3')	Primer sequences	Amplicon size (bp)	Source
**Human**				
HPRT1	Sense	TGACACTGGCAAAACAATGCA	94	Primer3
	Anti-sense	GGTCCTTTTCACCAGCAAGCT		
SIRT5	Sense	CCTGTGGAACGACTCTTCCT	182	Primer3
	Anti-sense	GCGAGGGAATCAGAGGCTAT		
**Mouse**				
Kim1	Sense	TCAGCTCGGGAATGCACAA	67	Honaparish et al., 2018[2]
	Anti-sense	TGGTTGCCTTCCGTGTCTCT		
Actin	Sense	GATTCCATACCCAAGAAGGAAGGC	128	Kipp et al., 2013[3]
	Anti-sense	CACTGCCGCATCCTCTTCCT		
B2M nDNA	Sense	TGCTGTCTCCATGTTTGATGTATCT	86	Bai and Wong, 2005 [4]
	Anti-sense	TCTCTGCTCCCCACCTCTAAGT		
mtDNA D-loop	Sense	CATCTGGTTCCTACTTCAGGG	104	
	Anti-sense	TGAGTGGTTAATAGGGTGATAGA		

Table S2. Antibodies for Western blot analyses.

Name	Species	Clonality (clone)	Supplier	Cat#	Dilution
**Loading controls**					
β-Actin	rabbit	pc	SIGMA-ALDRICH	A2066	1:5000
β-Actin	mouse	mc (C4)	SANTA CRUZ	sc-47778	1:3000
α-Tubulin	rabbit	mc (EP1332Y)	abcam	ab52866	1:500,000
**SIRT5**					
SIRT5	rabbit	pc	ATLAS ANTIBODIES	HPA022002	1:2000
SIRT5	rabbit	mc (D8C3)	Cell Signalling	8782	1:1000
**Autophagy-associated proteins**					
LC3-I/II	rabbit	pc	Cell Signalling	4108	1:1000
LC3-I/II	rabbit	pc	Cell Signalling	2775	1:1000
p62	rabbit	pc	Cell Signalling	5114	1:1000
**Mitochondrial marker proteins**					
SDHA	mouse	mc (F-2)	SANTA CRUZ	sc-390381	1:1000
TIM23	mouse	mc (H-8)	SANTA CRUZ	sc-514463	1:1000
TOM20	mouse	mc (F-10)	SANTA CRUZ	sc-17764	1:1000
TOM20	rabbit	pc	proteintech	11802-1-AP	1:3000
VDAC1	mouse	mc (B-6)	SANTA CRUZ	sc-390996	1:1000
**Mitochondrial fusion-associated proteins**					
MFN1	mouse	mc (3C9)	abcam	ab57602	1:1000
MFN2	mouse	mc (6A8)	abcam	ab56889	1:1000
OPA1	mouse	mc (18/OPA1)	BD Biosciences	612606	1:1000
**Mitochondrial fission-associated proteins**					
DRP1	mouse	mc (C-5)	SANTA CRUZ	sc-271583	1:1000
DRP1-S616	rabbit	pc	Cell Signalling	8570	1:1000
FIS1	mouse	mc (B-5)	SANTA CRUZ	Sc-376447	1:1000
MiD51	rabbit	pc	proteintech	20164-1-AP	1:3000
OMA1	mouse	mc (H-11)	SANTA CRUZ	sc-515788	1:1000
YME1L	rabbit	pc	proteintech	11510-1-AP	1:1000
**Apoptosis marker**					
cCASP3	rabbit	pc	Cell Signalling	9662	1:1000

Key: mc: monoclonal, pc: polyclonal.

Table S3. Antibodies for Immunohistochemistry and Immunofluorescence.

Name	Species	Clonality (clone)	Supplier	Cat#	Dilution
**Primary antibodies**					
AQP1	mouse	mc (1/22)	abcam	ab9566	IF (1:3000)
Calbindin-D-28k	mouse	mc (CB-955)	SIGMA-ALDRICH	C9848	IF (1:3000)
SIRT5	rabbit	pc	ATLAS ANTIBODIES	HPA022002	IHC/IF (1:200)
SIRT5	rabbit	mc (D8C3)	Cell Signalling	8782	IHC/IF (1:200)
IgG control	rabbit	pc	R&D Systems	AB-105-C	
IgG control	mouse	mc	R&D Systems	MAB002	
**Secondary antibodies**					
Goat anti-mouse IgG (H+L), Alexa Fluor 555	goat	pc	Thermo Fisher Scientific	A-21422	IF (1:500)
Goat-anti-rabbit IgG (H+L) (AlexaFluor 488)	goat	pc	abcam	ab150081	IF (1:500)

Key: H+L: Heavy and light chains, IF: Immunofluorescence, IHC: Immunohistochemistry, mc: monoclonal, pc: polyclonal.

Supplementary References

1 Hatefi, Y. & Stiggall, D. L. Preparation and properties of succinate: ubiquinone oxidoreductase (complex II). *Methods Enzymol.* **53**, 21-27, doi:10.1016/s0076-6879(78)53008-5 (1978).

2 Honarpisheh, M. *et al.* Aristolochic acid I determine the phenotype and activation of macrophages in acute and chronic kidney disease. *Sci Rep* **8**, 12169, doi:10.1038/s41598-018-30628-x (2018).

3 Kipp, A. P., Frombach, J., Deubel, S. & Brigelius-Flohé, R. Selenoprotein W as biomarker for the efficacy of selenium compounds to act as source for selenoprotein biosynthesis. *Methods Enzymol* **527**, 87-112 (2013).

4 Bai, R. K. & Wong, L. J. Simultaneous detection and quantification of mitochondrial DNA deletion(s), depletion, and over-replication in patients with mitochondrial disease. *J Mol Diagn* **7**, 613-622, doi:10.1016/S1525-1578(10)60595-8 (2005).
